# Lack of IRF6 Disrupts Human Epithelial Homeostasis by Altering Colony Morphology, Migration Pattern, and Differentiation Potential of Keratinocytes

**DOI:** 10.3389/fcell.2021.718066

**Published:** 2021-09-30

**Authors:** Eleftheria Girousi, Lukas Muerner, Ludovica Parisi, Silvia Rihs, Stephan von Gunten, Christos Katsaros, Martin Degen

**Affiliations:** ^1^Laboratory for Oral Molecular Biology, Dental Research Center, Department of Orthodontics and Dentofacial Orthopedics, University of Bern, Bern, Switzerland; ^2^Institute of Pharmacology, University of Bern, Bern, Switzerland

**Keywords:** IRF6, oral mucosa, skin, differentiation, wound healing, proteomics, GRHL3

## Abstract

Variants within the gene encoding for the transcription factor Interferon Regulatory Factor 6 (IRF6) are associated with syndromic and non-syndromic Cleft Lip/Palate (CLP) cases. IRF6 plays a vital role in the regulation of the proliferation/differentiation balance in keratinocytes and is involved in wound healing and migration. Since a fraction of CLP patients undergoing corrective cleft surgery experience wound healing complications, *IRF6* represents an interesting candidate gene linking the two processes. However, Irf6 function has been mainly studied in mice and knowledge on IRF6 in human cells remains sparse. Here, we aimed to elucidate the role of IRF6 in human postnatal skin- and oral mucosa-derived keratinocytes. To do so, we applied CRISPR/Cas9 to ablate IRF6 in two TERT-immortalized keratinocyte cultures, which we used as model cell lines. We show that IRF6 controls the appearance of single cells and colonies, with the latter being less cohesive in its absence. Consequently, IRF6 knockout keratinocytes often moved as single cells instead of a collective epithelial sheet migration but maintained their epithelial character. Lack of IRF6 triggered severe keratinocyte differentiation defects, which were already apparent in the stratum spinosum and extended to the stratum corneum in 3D organotypic skin cultures, while it did not alter their growth rate. Finally, proteomics revealed that most of the differentially expressed proteins in the absence of IRF6 could be associated with differentiation, cell-cell adhesion as well as immune response. Our data expand the knowledge on IRF6 in human postnatal keratinocytes, which will help to better understand IRF6-related pathologies.

## Introduction

Interferon regulatory factor 6 (IRF6) belongs to a family of nine transcription factors that mediate the expression of interferon following viral infections (Yanai et al., 2012). In contrast to the other family members, which are strictly involved in innate and adaptive immune processes (Ikushima et al., 2013), IRF6 has been found to be essential for proper craniofacial morphogenesis and skin homeostasis (Ingraham et al., 2006). In humans, rare *IRF6* variants are causal for Van der Woude syndrome (VWS, OMIM: 119300) and Popliteal Pterygium syndrome (PPS, OMIM: 119500), which are characterized by the presence of orofacial clefts, lip pits as well as cutaneous and limb defects. In addition, *IRF6* variants have also been found associated with isolated, non-syndromic orofacial clefts (Kondo et al., 2002; Zucchero et al., 2004; Leslie et al., 2013).

Mouse models, such as a total *Irf6* knockout (Ingraham et al., 2006) as well as a mouse harboring an *Irf6^*R*84*C*^*^/^*^*R*84*C*^* variant (Richardson et al., 2006), have been pivotal in the understanding of IRF6 function. *Irf6* disruption in mice results in perinatal lethality associated with severe skin, limb, and craniofacial anomalies (Ingraham et al., 2006). In accordance with these observations and the clinical VWS/PPS phenotypes, *Irf6* was found to be broadly expressed in embryonic and adult murine tissues with highest levels in the fusing palatal shelves, hair follicles, palatal rugae, tooth germs and thyroglossal duct, external genitalia, and skin (Kondo et al., 2002; Knight et al., 2006). The main role of IRF6 has been attributed to its function as a master regulator of the balance between keratinocyte proliferation and differentiation. Indeed, *Irf6* knockout mice exhibit a hyperproliferative epidermis with aberrant localization of proliferating keratinocytes in the suprabasal spinous cell layer. Concomitantly, epidermal keratinocytes fail to undergo terminal differentiation and lack a functional periderm, a second cell layer that covers the embryonic epithelia and protects them from pathological adhesions (Ingraham et al., 2006; Richardson et al., 2006, 2014; Hammond et al., 2019). Such premature oral adhesions are believed to hinder palatal shelf elevation during palatogenesis, resulting in orofacial clefts (Richardson et al., 2014). All these seminal findings in animal models were more recently complemented and extended by elucidating the intrinsic cellular behavior of embryonic murine *Irf6*^–/–^ keratinocytes *in vitro*. Similar to the *in vivo* situation, the balance between keratinocyte proliferation and differentiation was found to be altered in *Irf6*^–/–^ keratinocytes, as they lack the capacity to terminally differentiate and display an increased long-term proliferative potential compared to their wildtype (wt) counterparts (Biggs et al., 2012). Notably, lack of Irf6 also resulted in an abnormal keratinocyte appearance *in vitro* with many cells being larger than controls and presenting with an increased network of stress fibers (Biggs et al., 2012, 2014).

Successful cutaneous wound healing depends on a well-orchestrated series of cellular events such as proliferation, migration, and differentiation leading to the repair of tissue damage (Shaw and Martin, 2009). Similar cellular processes are also required for the morphogenesis of the secondary palate during embryogenesis (Bush and Jiang, 2012; Lan et al., 2015). Therefore, it has been hypothesized that palatogenesis and wound healing share common genes and pathways for their distinct, but similar accomplishment of closing a tissue gap and forming a seal (Biggs et al., 2015). *IRF6* might represent such a candidate gene. Indeed, particular wound healing defects like reduced speed and directionality during wound re-epithelialization, as well as impaired maturation of the granulation tissue, have been described in embryonic *Irf6*^–^*^/^*^–^ keratinocytes *in vitro* (Biggs et al., 2014) and in *Irf6*^+^*^/^*^–^ mice *in vivo* (Rhea et al., 2020), respectively. These observations might also provide the molecular rationale for the increased likelihood of wound healing complications experienced by VWS patients harboring *IRF6* variants in comparison to non-syndromic cleft patients (Jones et al., 2010).

In the last decades, considerable knowledge has been gained about IRF6 function during craniofacial development, mostly using either *in vivo* animal models or embryonic keratinocytes, derived from *Irf6*^–^*^/^*^–^ mice. Still, only a handful of transcriptional targets of IRF6, such as *GRHL3*, *OVOL1* and *KLF4*, have been identified and described so far (Botti et al., 2011; de la Garza et al., 2013; Liu et al., 2016). Although a role for IRF6 in murine keratinocyte migration and embryonic wound healing has been reported, little is known about IRF6 function in postnatal tissue repair in human cells. Since epithelial-specific differences in wound healing have been established and described (Eming et al., 2014; Turabelidze et al., 2014), we sought to decipher the role of IRF6 in human postnatal keratinocytes isolated from two different sources: oral mucosa and foreskin.

We used a CRISPR/Cas9 approach to generate IRF6 knockout keratinocytes derived from postnatal oral mucosa and skin tissue, which allowed us to study IRF6 function in two distinct and relevant tissue contexts. We supplemented this approach with a proteomic analysis to discover novel potential targets or interactors of IRF6. While our data confirm certain previous findings in murine models, there are some differences, which might be specific to human cells. In addition, we also reveal that all the phenotypes in response to IRF6 ablation were present in skin- and oral mucosa-derived keratinocytes, although proteomics reported significantly less changes in the absence of IRF6 in oral mucosal keratinocytes. Our study substantially expands the knowledge of IRF6 function in postnatal human keratinocytes and will be important for a better understanding of VWS and/or general orofacial cleft-related complications or other IRF6-related pathologies.

## Materials and Methods

### Primary Cell Isolation

Foreskin tissue samples were obtained from two to five years old healthy boys during routine circumcision at the Children’s Hospital, University of Bern. Oral mucosa samples were received from non-syndromic (no mutation within *IRF6*) CLP patients at the age of 3-6 months during corrective surgery to close the cleft lip. From one CLP patient, we were able to get both oral mucosa as well as skin tissue.

Primary keratinocytes were isolated from the tissue samples using the explant culture system as described elsewhere (Degen et al., 2018). After their outgrowth, keratinocytes were purified from contaminating fibroblasts by differential trypsinization, followed by their expansion in keratinocyte serum-free medium (KSFM, Gibco, Thermo Fisher Scientific, Lucerne, Switzerland) supplemented with 25 μg/ml bovine pituitary extract, 0.2 ng/ml epidermal growth factor (EGF), and CaCl_2_ to a final Ca^2+^ concentration of 0.4 mM, as previously described (Degen et al., 2013; Parisi et al., 2021). All experiments using primary cells have been performed with cultures from the second to the fourth passage.

### Cell Culture and Treatments

The immortalized oral mucosal keratinocytes OKF6/TERT2 (derived from the floor of the mouth) as well as the immortalized foreskin-derived strain N/TERT1 keratinocytes (Dickson et al., 2000) were cultured in complete KSFM.

For growth factor treatments, keratinocytes were grown to 60% confluency followed by addition of EGF (Thermo Fisher Scientific), Transforming Growth Factor β1 and β3 (TGFβ1, TGFβ3, PeproTech, London, United Kingdom) at the indicated concentrations and times before harvesting either RNA or protein samples.

### Immunoblotting

Whole protein lysates from cells were prepared in RIPA buffer (10 mM Tris-HCl (pH 8.0), 1 mM EDTA, 0.1% sodium deoxycholate, 0.1% SDS, 1% NP40, 140 mM NaCl) supplemented with cOmplete Mini^TM^ Protease Inhibitor cocktail and PhosSTOP EASYpack (both from Sigma-Aldrich, St. Louis, MO, United States). The BCA Protein Assay Kit (Pierce, Thermo Fisher Scientific) was used to measure protein concentrations of the samples. Approximately 20 μg of protein were mixed with loading buffer (2% SDS, 62.6 mM Tris-HCl, pH 6.8, 10% glycerol, 0.01% bromophenol blue) containing 100 mM dithiothreitol (DTT), boiled for 5 min at 95°C, fractionated by SDS-PAGE under reducing conditions and transferred to nitrocellulose membranes (Sigma-Aldrich). The membranes were then stained with 0.1% amido black solution (MERCK, Schaffhausen, Switzerland) to assess blotting efficiency and equal protein loading. After extensive washing, the membranes were blocked for 1 h at room temperature (RT) in 5% skim milk powder (Sigma-Aldrich) dissolved in Tris-buffered saline (pH 7.4) with 0.05% Tween (TBS-Tween), and then incubated with primary antibodies overnight at 4°C. Blots were washed three times in TBS-Tween followed by incubation with horseradish peroxidase-conjugated anti-mouse/rabbit IgG (Thermo Fisher Scientific) for 1 h at RT. After three more washes in TBS-Tween, blots were developed using SuperSignal West Dura or West Pico (Thermo Fisher Scientific) and scanned by an Imager Chemi Premium Imager Instrument (VWR, Darmstadt, Germany).

Primary antibodies used for immunoblots: Rabbit polyclonal antibodies anti-Fibronectin (Wehrle-Haller et al., 1991), anti-E-Cadherin (20874-1-AP, Proteintech, Manchester, United Kingdom), anti-TGM1 (Thermo Fisher Scientific), and anti-GRHL3 (ARP39489_T100; Aviva Systems Biology, San Diego, CA, United States). Mouse monoclonal antibodies anti-IRF6 (14B2C16, BioLegend, San Diego, CA, United States), anti-Involucrin (SY5, BIO-RAD, Hercules, CA, United States), anti-Vinculin (V9131, Sigma-Aldrich).

Some of the immunoblots were densitometrically analyzed using the ImageJ software^1^. Briefly, the intensity of each protein band was normalized to the vinculin band intensity of the same extract in the same experiment.

### Immunofluorescence

For immunofluorescent staining, keratinocytes were cultured in 35 mm dishes containing four separate wells (Greiner Bio-One, Frickenhausen, Germany). Cells were washed twice with phosphate-buffered saline (PBS) before fixation in 4% paraformaldehyde (PFA) for 20 min at RT. Fixation was followed by three washing steps in PBS, permeabilization in 0.1% Triton-X-100 for 5 min, and incubation with primary antibody for 2 h at RT in PBS/3% bovine serum albumin. Afterward, keratinocytes were extensively washed with PBS and incubated with fluorescent-labeled secondary goat anti-mouse/rabbit IgG (Molecular Probes, Thermo Fisher Scientific) and/or tetramethylrhodamine (TRITC)-phalloidin (Sigma-Aldrich) for 1 h in the dark. Finally, cells were washed three times with PBS and once with H_2_O before being coverslip-mounted with Vectashield Mounting Medium containing DAPI (Vector Laboratories, Burlingame, CA, USA).

Analysis was performed using an Olympus BX-51 phase/fluorescence microscope (OlympusLife Science Solutions, Tokyo, Japan) equipped with a xenon lamp (X-Cite, series 120PC Q, Lumen Dynamics, Mississauga, Canada). Images were captured by a ProgRes CT3 camera with ProgRes CapturePro software (Jenaoptik, Jena, Germany), using a 20x/0.5 objective.

Primary antibodies used for immunofluorescent staining: Rabbit polyclonal antibodies anti-IRF6 (NBP2-49383, Novus Biologicals, Centennial, CO, United States), anti-E-Cadherin (20874-1-AP, Proteintech), anti-TGM1 (NBP2-34062, Novus Biologicals), and anti-Loricrin (PA5-30583, Thermo Fisher Scientific). Mouse monoclonal antibody anti-Involucrin (SY5, BIO-RAD).

### Proteomic Analysis

Keratinocytes were grown to high density before protein extraction in 8M urea/100 mM Tris. Reduction, alkylation and precipitation steps were performed overnight. Protein pellets were then resuspended in 8M urea/50mM Tris pH8 and their concentrations were determined with the Qubit Protein Assay (Invitrogen, Thermo Fisher Scientific). 10 μg of protein were digested with LysC for 2 h at 37°C, followed by Trypsin digestion overnight at RT.

The digests were analyzed by liquid chromatography on a Dionex, Ultimate 3,000 coupled to a LUMOS mass spectrometer (Thermo Fisher Scientific) with two injections of 500 ng peptides. The samples were loaded in random order onto a pre-column (C18 PepMap 100, 5 μm, 100 Å, 300 μm i.d. × 5 mm length) at a flow rate of 50 μL/min with solvent C (0.05% TFA in water/acetonitrile 98:2). After loading, peptides were eluted in back flush mode onto a C18 column (5 μm, 100 Å, 75 μm × 15 cm) by applying a 90-min gradient of 5 to 40% acetonitrile in water, 0.1% formic acid, at a flow rate of 400 nl/min. Data acquisition was made in data dependent mode with precursor ion scans recorded in the orbitrap with resolution of 120’000 (at m/z = 250) parallel to top speed fragment spectra of the most intense precursor ions in the linear trap for a cycle time of 3 s maximum.

Data were processed with MaxQuant (version 1.6.14.0) against the Homo Sapiens swissprot database (release October 2020) using default settings for peak detection, strict trypsin cleavage rule allowing a maximum of three missed cleavages. Carbamidomethylation on cysteine was set as a fixed modification, methionine oxidation and protein N-terminal acetylation as variable modifications.

Protein intensities were reported as MaxQuant’s Label Free Quantification (LFQ) values, as well as iTop3 (top3) values (sum of the intensities of the three most intense peptides). For the latter, variance stabilization (vsn) was used for the peptide normalization, and missing peptide intensities were imputed in the following manner: if there was at most one evidence in the group of replicates, the missing values were drawn from a Gaussian distribution of width 0.3 centered at the sample distribution mean minus 2.5× the sample’s standard deviation, otherwise the Maximum Likelihood Estimation (MLE) method was used. Imputation at protein level for LFQ was performed if there were at least two measured intensities in at least one group of replicates. In this case, missing values were drawn from a Gaussian distribution as described before if there was at most one evidence in the replicate group, otherwise MLE was used.

Evaluation of overrepresented Gene Ontology (GO) biological processes was performed using Panther^2^. To display protein interactions, selected proteins were uploaded into String database^3^. Venn diagrams were designed using the jvenn software^4^.

### RNA Extraction, cDNA Synthesis and Quantitative Real-Time Polymerase Chain Reaction (qPCR)

Total RNA was purified from cells using the innuPREP RNA Mini kit (Analytik Jena AG, Jena, Germany) according to the standard protocol for eukaryotic cells. RNA concentration and quality was assessed using a Nanodrop 2000c (Thermo Fisher Scientific).

500 ng of total RNA were used as template for cDNA synthesis with M-MLV Reverse Transcriptase and Oligo(dT)_15_ Primer (both from Promega, Dübendorf, Switzerland). The analysis and quantification of the mRNA levels were performed by qPCR using GoTaq qPCR Master Mix (Promega) on a QuantStudio 3 instrument (Applied Biosystems, Thermo Fisher Scientific). The ΔC_*T*_ or the ΔΔC_*T*_ method was used for the calculation of the mRNA expression, normalizing values of each sample to *GAPDH*. qPCR primers (Supplementary Table 1) were designed with the NCBI primer tool^5^ and tested for specificity and efficiency.

### Lentiviral Plasmids and Virus Preparation

Lentiviruses were produced in HEK293T cells with the packaging vectors psPAX2 and the pMD2.G plasmid (both gifts from Didier Trono (Addgene plasmids #12260 and 12259)). The pLentiCas9-BFP and the pDECKO_mCherry plasmids (both gifts from Roderic Guido & Rory Johnson (Addgene plasmids #78545 and 78534)) were used for the CRISPR knockouts, while pCAD-IRES-GFP was applied for the rescue experiments. 24 h after cell transfection with ViaFect (Promega) in OptiMEM, transfection medium was replaced with Dulbecco’s modified Eagle’s medium (DMEM) (Thermo Fisher Scientific) containing 10mM HEPES, pH7.4 (Thermo Fisher Scientific). Virus-containing supernatants were collected 48 and 72 h after transfection, pooled and sterile-filtered.

### IRF6 CRISPR/Cas9 Knockout Keratinocytes

Keratinocytes were transduced in 6-well plates by spinfection (2,000 rpm for 90 min at 37°C) in the presence of 5 μg/ml polybrene (Sigma-Aldrich) with the pLentiCas9-BFP viral supernatant. After transduction, Cas9-positive cells were selected for with 4 μg/ml Blasticidine S hydrochloride (Sigma-Aldrich) and sorted twice for BFP expression using the MoFlo^®^ Astrios^TM^ EQ cell sorter (Beckman Coulter Life Sciences, Krefeld, Germany).

To target *IRF6*, two single guide RNAs (sgRNA) were designed specific for IRF6 exon number 3 (5′-TGGGCTCATCTGGCTACACA-3′) and exon 4 (5′-CCCTGACCCAGCTAAATGGA-3′), respectively, using CRISPETa (CRISPR Paired Excision Tool) software pipeline. The oligos were purchased from Microsynth AG (Balgach, Switzerland) and cloned into the pDECKO_mCherry vector using Gibson Assembly Master Mix (New England Biolabs, Ipswich, MA, United States). Plasmid was sequence-verified before production of the lentiviral supernatant. 5 × 10^4^ Cas9 expressing NTERT/1 cells were seeded in 12-well plates and transduced with lentivirus suspension (pDECKO_mCherry containing the two IRF6 sgRNAs) diluted 1:1 in DMEM in the presence of 5 mg/ml polybrene using spinfection. Medium was changed 4-6 h after the transduction to KSFM containing HEPES buffer. In contrast, OKF6/TERT2 keratinocytes were transiently transfected with the pDECKO_mCherry plasmid containing the two IRF6-specific guide RNAs using ViaFect (Promega). For both approaches, mCherry-positive cells were sorted into 96-well plates as single cells, expanded, and the IRF6 knockout was verified in the selected clones by Sanger DNA sequencing, staining, and immunoblotting.

### Crystal Violet Staining and Cell Morphology Analysis

Morphology of single cells and of colonies was assessed by crystal violet staining. Briefly, cells were fixed in 4% PFA for 20 min at RT, washed in PBS and stained with 0.5% crystal violet (Sigma-Aldrich) in 20% methanol for 20 min at RT. Excessive stain was removed by rinsing the dishes extensively with H_2_O before air-drying. Representative pictures of the cell colonies were taken using the Olympus BX-51 phase microscope (OlympusLife Science Solutions).

The ImageJ software was used to quantify the cell size and the cell colony circularity. Circularity was calculated as c = 4π(A/P^2^). A: area of colony; P: perimeter of colony. For a perfect circle: c = 1. Analysis was performed blinded by two members of the laboratory (LP and MD).

### Scratch Assay

Keratinocytes were cultured until confluence. The monolayer was scratched using a sterile 20 μl pipette tip. Cells were then washed with PBS to remove cell debris and incubated in fresh medium at 37°C/5% CO_2_. Images of identical spots of the scratches were captured every hour. Closure of the scratch was analyzed using the TScratch software^6^.

Alternatively, we used the IncuCyte S3 (Sartorius, Göttingen, Germany) live imaging device. Briefly, 6 × 10^4^ keratinocytes were seeded in a 96-well ImageLock^TM^ Microplate (Sartorius). After reaching confluency, the monolayer was scratched with a 96-pin Incucyte WoundMaker (Sartorius). Scratch assays were analyzed with the Cell Migration Software Application Module (Sartorius).

### Cell Growth

To determine cell growth, 10^4^ cells/ml were seeded into 60 mm culture dishes. After attachment, cells were trypsinized and counted (*t* = 0) using a Neubauer Counting chamber and an automated cell counter (Countess^TM^ II, Invitrogen, Thermo Fischer Scientific) using Trypan Blue as a viability marker. KSFM was replaced every other day and keratinocytes counted daily for 5 days.

### *In vitro* Differentiation

Keratinocytes were grown in basal KSFM (0.1 mM CaCl_2_) for 3 days. Thereafter, 6 × 10^4^ cells were seeded into 35 mm culture dishes in basal KSFM. 24 h later, Ca^2+^ concentration was increased to 1.2 mM to induce differentiation. 3 days after this Ca^2+^-switch, total RNA and proteins were extracted and parallel cultures were fixed.

Alternatively, we applied a cell density-dependent differentiation assay. For that purpose, keratinocytes were grown in fully supplemented KSFM and plated at a cell density of 10^5^ cells/100 mm dish. After the emergence of first colonies (2-3 days), RNA and protein samples were extracted, and parallel dishes fixed (low density LD). The same samples were collected of parallel cultures at full confluence (high density HD).

### 3D-Skin Models

For the 3D-skin models of control and IRF6 KO keratinocytes, the protocol from CELLnTEC (CELLnTEC advanced cell system AG, Bern, Switzerland) was used. Briefly, 2 × 10^5^ keratinocytes were seeded in 400 μl KSFM into polycarbonate inserts (0.4 μm pore size, 12 mm diameter, Nunc, Thermo Fisher Scientific) placed in 60 mm tissue culture dishes, immediately followed by the addition of 11 ml of KSFM outside the inserts. Confluency was confirmed by staining one insert from each culture with the staining kit (CnT-ST-100, CELLnTEC). In confluent monolayer cultures, differentiation of keratinocytes was induced by switching from KSFM to 3D Barrier Medium (CnT-PR-3D, CELLnTEC), added both inside and outside of the insert (equal level) overnight. After 24 h, the medium inside the insert was completely removed and the one outside of the insert was replenished with 3.2 ml 3D Barrier Medium, lifting the membranes to the air-liquid interface. Thereafter, keratinocytes were incubated for 15 days at 37°C/5% CO_2_ with medium replacement 3 times per week. 3D-cultures were then fixed in 4% PFA for 2 h at 4°C. The polycarbonate membranes were excised from the inserts, cut into two pieces, placed in embedding cassettes between two biopsy pads, and stored in 0.1 M sodium cacodylate buffer at 4°C. Membranes were dehydrated, paraffin embedded and sectioned on a Reichert-Jung microtome (Leica Microsystems, Heerbrugg, Switzerland). Paraffin sections were deparaffinized, rehydrated through xylene, ethanol, and deionized H_2_O, and stained with hematoxylin and eosin (H&E). H&E sections were analyzed and quantified using the ImageJ software.

Immunohistochemical (IHC) staining reactions were performed by automated staining using a BOND RX autostainer (Leica Microsystems). Briefly, sections were deparaffinized and antigen was retrieved using 1 mM Tris solution (pH 9.0) for 30 min at 95°C. Sections were stained with primary rabbit polyclonal anti-Loricrin antibody (PA5-30583; Thermo Fisher Scientific) followed with secondary antibody, and specific binding of primary antibodies was visualized using a polymer-based visualizing system with horseradish peroxidase as the enzyme and 3,3-diaminobenzidine (DAB) as a brown chromogen (Leica Microsystems). Finally, the samples were counterstained with hematoxylin and mounted with Aquatex (Merck, Burlington, MA, United States).

### Generation of IRF6/GRHL3 Overexpressing Cells

Full-length cDNAs encoding for human IRF6 and GRHL3 were cloned into the lentiviral expression vector CAD-IRES-GFP by Genescript (Leiden, Netherlands). Lentiviral supernatants and transductions were prepared as described before. Transduced keratinocytes were sorted for GFP expression and the population of GFP-positive cells (pool) was used for rescue experiments.

### Statistical Analysis

Experiments were performed at least three times in multiple replicates. Data were analyzed using Prism 7.0 (GraphPad, La Jolla, CA, United States). Data are represented as means ± standard deviation (SD). Multiple comparisons were performed using one- or two-way analysis of variance (ANOVA) with Tukey’s *post hoc* test. Values of *p* ≤ 0.05 were considered significant.

## Results

### Higher Expression of IRF6 in Skin- Compared to Oral Mucosa-Derived Keratinocytes

**FIGURE 1:**
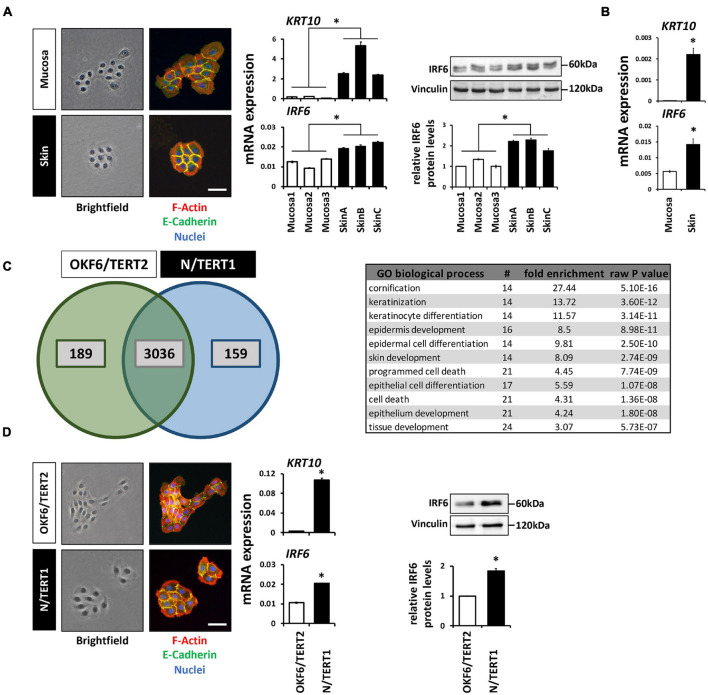
**(A)** Comparison of the colony morphology, *Keratin10* (*KRT10*) and IRF6 expression in three individual primary oral mucosa-derived keratinocytes (Mucosa 1-3) compared to three foreskin-derived keratinocytes (Skin A-C). Note that the skin-derived cells form tightly packed colonies, express high levels of *KRT10*, and express more IRF6 than mucosa-derived cells. ^∗^*p* < 0.05 (IRF6 and *KRT10* levels in Skin vs. Mucosa). Scale bar: 100 μm. kDa: kilo Dalton. **(B)** *KRT10* and *IRF6* mRNA levels of mucosal- and skin-derived keratinocytes from the same donor show higher expression of both genes in the skin. ^∗^*p* < 0.05 (*KRT10* and *IRF6* levels in Skin vs. Mucosa). **(C)** Venn diagram of the proteomic analysis comparing OKF6/TERT2 (oral mucosa) and N/TERT1 (foreskin) keratinocytes reveals a high number of proteins present in both cell lines, 189 and 159 proteins are unique to OKF6/TERT2 and N/TERT1, respectively. From the 3,036 shared proteins, 91 proteins are differentially expressed (>2 fold) and mostly associated with the biological processes of epidermal differentiation, keratinization, and cornification (table right). **(D)** Similar to the observations in primary keratinocytes, N/TERT1 keratinocytes form more cohesive and regularly shaped colonies and express higher levels of *KRT10* and IRF6 than the oral mucosa-derived OKF6/TERT2. Scale bar: 100 μm. ^∗^*p* < 0.05 (*KRT10* levels in Skin vs. Mucosa); ^∗^*p* < 0.05 (IRF6 levels in Skin vs. Mucosa). Full-length immunoblots are shown in Supplementary Figure 9.

Next to immune cells (Joly et al., 2016) and certain osteocytes (Thompson et al., 2019), IRF6 is greatly expressed in cutaneous and oral keratinocytes (Ingraham et al., 2006; Knight et al., 2006). We took advantage of our primary cell bank (Parisi et al., 2021) and compared the morphology, levels of differentiation markers, and IRF6 levels between three oral mucosa- and three independent foreskin-derived keratinocyte populations. Tissue-specificity of the keratinocyte cultures (at the same cell density) was distinguishable from each other since skin-derived keratinocyte formed coherent and round-shaped colonies whereas the ones from oral mucosa keratinocytes appeared polymorph and less compact, and since only skin keratinocytes were found to display robust Keratin10 (*KRT10*) levels. In regard to IRF6, we detected a significantly higher mRNA and protein expression in the skin-derived than in the mucosa-derived keratinocytes (Figure 1A). From one additional tissue donor, we were able to isolate both skin- and mucosa-derived keratinocytes. We used these cells to prove that the observed difference in IRF6 expression between mucosal- and skin-derived keratinocytes (Figure 1A) is not due to the variability among tissue donors. After confirming tissue identity of the cells by the presence or absence of robust *KRT10* levels, we observed an increased *IRF6* mRNA expression in skin keratinocytes when compared to the oral mucosa counterpart (Figure 1B). These distinct IRF6 expression levels suggest certain tissue-specific activities of IRF6. This prompted us to address IRF6 function in oral mucosa and skin keratinocytes by depleting it using a CRISPR/Cas9 approach.

We initially planned to use primary keratinocytes for the study, but refrained from this idea as the CRISPR/Cas9 approach requires several experimental steps (transductions, cell sorting, single cell outgrowths) that might be challenging to perform with primary keratinocytes, which are delicate to keep as healthy-growing (not-differentiated and not-senescent) cells over several passages. Envisioning these caveats when using primary keratinocytes for the CRISPR/Cas9 approach, we thought of alternative cell models reflecting the observations made in the primary cells. Therefore, we chose to use the well-established immortalized oral mucosal line OKF6/TERT2 (OKF6) and the foreskin-derived N/TERT1 (N) keratinocytes for future experiments (Dickson et al., 2000). First, we carefully characterized these two cell lines for their usefulness. Proteomics of the two cell lines cultured to high-density (HD) detected 3,036 proteins shared between them (Figure 1C). Of these common proteins, 91 were significantly differentially expressed (log_2_ fold change >1; Supplementary Figure 1 and Supplementary Table 2). These proteins mainly belonged to biological processes related to keratinization and cell differentiation, which confirms the mucosal- and skin-related origin of OKF6 and N keratinocytes, respectively. IRF6 was not included in the list of the differentially expressed proteins, but was found to be 1.5-fold higher expressed in N than in OKF6 keratinocytes. This was confirmed by qPCR showing higher *IRF6* mRNA expression in skin- than in in mucosa-derived keratinocytes (Figure 1D and Supplementary Data Sheet 2). In addition, OKF6 and N keratinocytes showed the typical colony morphology and *KRT10* expression pattern fitting to primary cells derived from their original tissues (Figure 1A). These observations as well as the fact that both TERT-immortalized keratinocyte lines are very similar to primary keratinocytes in regard to differentiation (Smits et al., 2017) let us conclude that OKF6 and N keratinocytes proved to be good cell models for our study.

### IRF6 Knockout in OKF6/TERT2 and N/TERT1 Keratinocytes

We applied the CRISPR/Cas9 approach to create IRF6 knockout OKF6 and N keratinocytes (Supplementary Figure 2A). Cell clones derived from single cells were thoroughly validated by DNA Sanger sequencing, immunoblotting and fluorescent staining for ablation of IRF6. Immunoblots for IRF6 confirmed absence of IRF6 in two OKF6 clones (#15 and #26) and in two N clones (#8 and #10), while the pool of the cells (bulk of cells after sorting upon guide RNA transfection/transduction) displayed strongly reduced IRF6 levels compared to their respective parental wildtype (wt) as well as stable Cas9-expressing cells (Supplementary Figure 2B). These results were further verified by staining for IRF6, which showed cytoplasmic expression of IRF6 in parental and Cas9-expressing cells, but is completely absent in the knockout cell clones (Supplementary Figure 2C).

### Lack of IRF6 Alters Cell-Cell Adhesion and Colony Morphology

**FIGURE 2:**
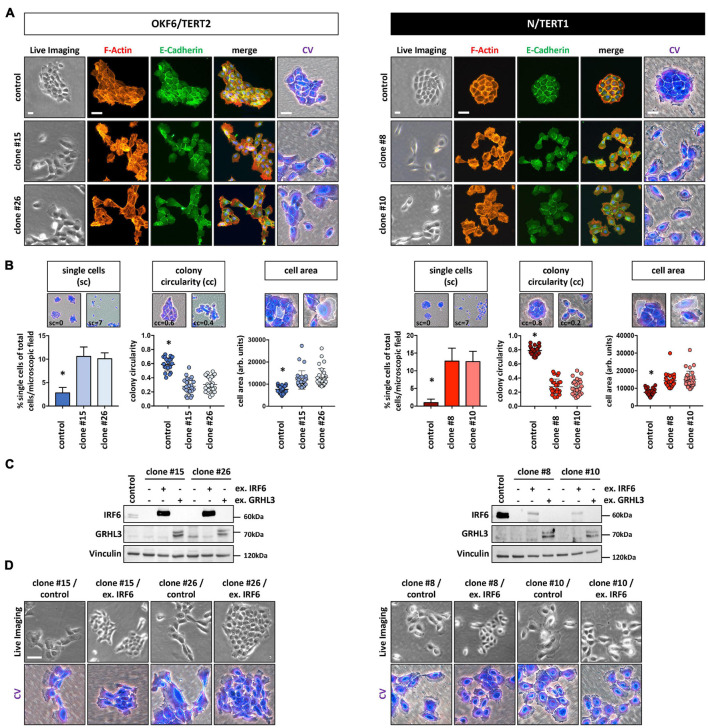
**(A)** In the absence of IRF6, OKF6/TERT2 and N/TERT1 keratinocytes change their colony morphology as assessed by live imaging, F-actin (phalloidin, red), E-Cadherin (green), and crystal violet (CV) staining. DAPI was used to stain for nuclei (blue). Scale bars: 20 μm (Live Imaging); 150 μm (F-Actin, E-Cadherin); 100 μm (CV). **(B)** IRF6 knockout in both OKF6/TERT2 and N/TERT1 cell lines results in significantly more single cells (sc), reduced colony circularity (cc) as well as in the emergence of larger single cells (cell area) when compared to their respective controls. ^∗^*p* < 0.05 controls vs. clones (sc); ^∗^*p* < 0.05 controls vs. clones (cc, cell area). **(C)** Immunoblots show overexpression of IRF6 and GRHL3 in the IRF6-deficient keratinocyte clones after transduction. Note the very little expression of ectopic IRF6 in N/TERT1 IRF6 knockout clones. kDa: kilo Dalton; ex.: exogenous. **(D)** Live imaging and crystal violet pictures of typical colony morphologies upon re-expression of IRF6 in the IRF6 K.O. clones. Note that low IRF6 levels are not able to rescue the dispersed colony phenotype. Scale bar: 100 μm. Full-length immunoblots are shown in Supplementary Figure 9.

Culturing the IRF6-deficient cells at low density (LD) revealed subtle, but significant differences at the single cell as well as cell colony morphology level. Proliferating OKF6 and N keratinocytes at LD formed cohesive and compact cell colonies with cells showing stable contacts, as assessed by live imaging, E-Cadherin positivity at the sites of cell-cell contacts, and crystal violet (CV) staining. In contrast, IRF6 ablation in keratinocytes impaired their capacity to form regularly shaped and coherent colonies (Figure 2A and Supplementary Figure 3). While we did not observe any obvious re-arrangements of the actin cytoskeleton (F-actin), IRF6-deficient keratinocyte colonies acquired a more scattered morphology with less stable expression of E-Cadherin at the cell-cell contacts. Often, single cells appeared to break free from the colony and we also observed an increased fraction of significantly enlarged cells in the cultures of IRF6-depleted keratinocytes compared to control (Figure 2A). In order to validate these observations, we analyzed 50 random pictures of CV-stained keratinocyte cultures at the same cell density for the presence of single cells as well as for their colony morphology (colony circularity) and single cell size (cell area). All these parameters were significantly altered in the IRF6 knockout clones compared to their corresponding controls. IRF6 ablation in both OKF6 and N keratinocytes resulted in the appearance of more single cells, a decreased colony circularity (less round colonies), and an increased proportion of enlarged cells (increased individual cell area) (Figure 2B). Prompted by these observations, we wanted to learn whether the observed cellular phenotype in the IRF6 knockout clones is due to IRF6 depletion and whether it can be rescued by either re-expressing IRF6 or one of its downstream targets, Grainyhead-like transcription factor 3 (GRHL3). Ectopic expression of IRF6 in IRF6-deficient cells resulted in strongly increased *IRF6* mRNA levels in both cell lines (Supplementary Figure 4A), but only to a robust overexpression of the IRF6 protein in OKF6 cells, with IRF6 remaining ablated in N IRF6-deficient cells (Figure 2C). This difference was due to the fact that the OKF6/Cas9 keratinocytes were transiently transfected with the IRF6-specific guide RNAs-containing vector, in contrast to its stable transduction into N/Cas9 keratinocytes (see Materials and Methods). In contrast, forced GRHL3 expression resulted in elevated GRHL3 mRNA and protein levels in both cell lines (Figure 2C and Supplementary Figure 4A). The phenotype of scattered cells and dispersed cell colonies in IRF6-deficient keratinocytes could be rescued by strong ectopic IRF6 in OKF6 clones, as these cells started to form cohesive colonies again. However, low IRF6 in N/TERT IRF6 knockout clones could not normalize the cell and colony morphology as assessed by live imaging and crystal violet staining (Figure 2D). GRHL3 may not have a role in colony morphology (Supplementary Figure 4B) but the effect of its overexpression in IRF6-deficient keratinocytes will be described later.

### IRF6-Deficient Keratinocytes Maintain Their Epithelial Character

**FIGURE 3:**
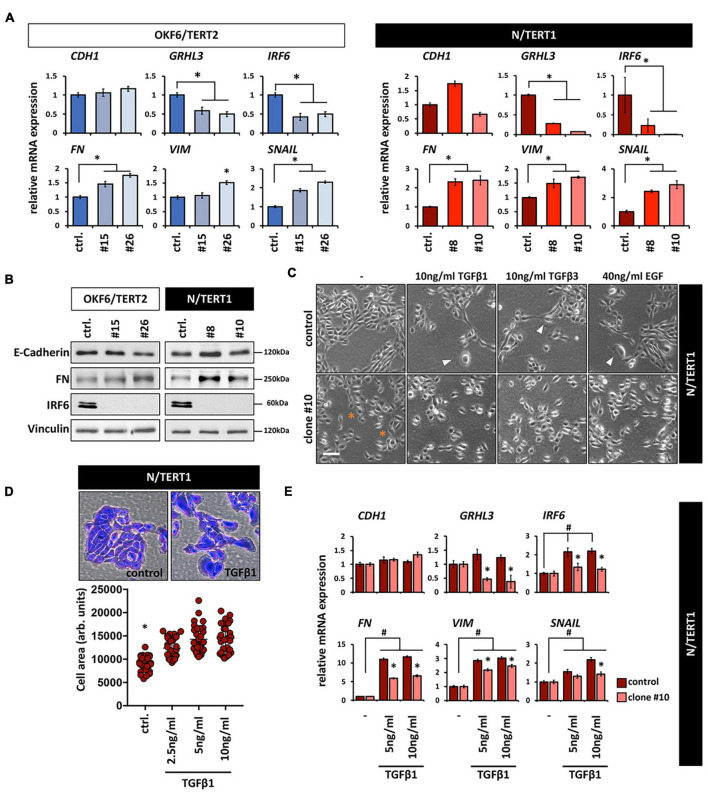
**(A)** qPCR analyses for the epithelial markers *CDH1*, *GRHL3*, and *IRF6* as well as for the mesenchymal markers *FN*, *VIM*, and *SNAIL* in OKF6/TERT2 (left) and N/TERT1 (right) cell lines. Note that although there is a slight increase of the mesenchymal markers, *CDH1* does not decrease in the absence of IRF6. ctrl.: control; ^∗^*p* < 0.05 controls vs. clones #15, #26, #8, #10. **(B)** Immunoblots confirm the qPCR results and show no change in E-Cadherin, but an increase in FN, and absence of IRF6 in the clones. kDa: kilo Dalton; ctrl.: control. **(C)** Treatment of N/TERT1 controls (top row) with the EMT-inducing growth factors EGF, TGFβ1, and TGFβ3 for 72 h induces changes in the cellular morphology with scattered and enlarged cells (arrowheads). In N/TERT1 clone #10 (bottom row) a similar morphological phenotype can already be appreciated without addition of growth factors. ^∗^indicates enlarged cells. Scale bar: 150 μm. **(D)** Crystal violet staining and cell area analysis of N/TERT1 control in the absence and presence of TGFβ1 (72 h) reveals the emergence of enlarged cells in the presence of the growth factor. ^∗^*p* < 0.05 control vs. TGFβ1-treated cells; ctrl.: control. **(E)** qPCR analyses for the mesenchymal markers *FN*, *VIM*, and *SNAIL* as well as the epithelial markers *CDH1*, *IRF6*, and *GRHL3* in TGFβ1-treated (72 h) cells in the presence (control) or absence (clone #10) of IRF6. Note that the mesenchymal markers as well as *IRF6* increase, while *CDH1* does not change. Also note that IRF6 is required for a proper modulation of all these markers. #*p* < 0.05 untreated vs. TGFβ1-treated cells; ^∗^*p* < 0.05 control vs. IRF6 knockout cells (clone #10). Full-length immunoblots are shown in Supplementary Figure 9.

Both the increased cell size and the appearance of more scattered and single cells are phenotypes that have been associated with epithelial-mesenchymal transition (EMT; (Lamouille and Derynck, 2007). This prompted us to test whether lack of IRF6 induces an EMT. IRF6-deficient keratinocytes showed a slight tendency of increased mRNA levels of the mesenchymal markers Fibronectin (*FN*), Vimentin (*VIM*), and Snail (*SNAIL*), while the epithelial markers *GRHL3* and *IRF6* were downregulated compared to the corresponding controls (Figure 3A). Notably, reduced *IRF6* mRNA levels are probably the results of either diminished stability of the altered transcripts or due to the missing IRF6 self-regulation (Botti et al., 2011) in IRF6-deficient cells. E-Cadherin (*CDH1*), one of the most prominent epithelial markers was not affected in response to IRF6 depletion (Figure 3A). Identical E-Cadherin levels, as well as altered levels of FN in the clones were confirmed by immunoblotting (Figure 3B). Although these results indicate that IRF6-defcient keratinocytes maintain their epithelial character, they were not conclusive yet in elucidating the role of IRF6 in the EMT process. Therefore, we triggered an EMT by treating N control and clone #10 keratinocytes with transforming growth factor β1 (TGFβ1), TGFβ3, and epidermal growth factor (EGF), which are known EMT inducing factors (Lamouille et al., 2014). Treatment of the control cells resulted in enlarged and more scattered cells, similar to the morphology of IRF6-deficient keratinocytes (Figures 3C,D) and in a robust and significant increase of the mesenchymal markers *FN*, *VIM* and *SNAIL*, suggestive of mesenchymal cell characteristics (Figure 3E). While TGFβ1 was not able to alter the levels of the epithelial markers *GRHL3* and *CDH1*, it triggered a significant increase of *IRF6* in control keratinocytes. This observation in combination with the fact that the induction of all mesenchymal markers was significantly impaired in the absence of IRF6 (clone #10) when compared to control suggests an important role for IRF6 as mediator during the regulation of EMT (Figure 3E) as it has been described in mice (Ke et al., 2015).

### IRF6 Is Required for Coordinated Movement of the Keratinocytes

**FIGURE 4:**
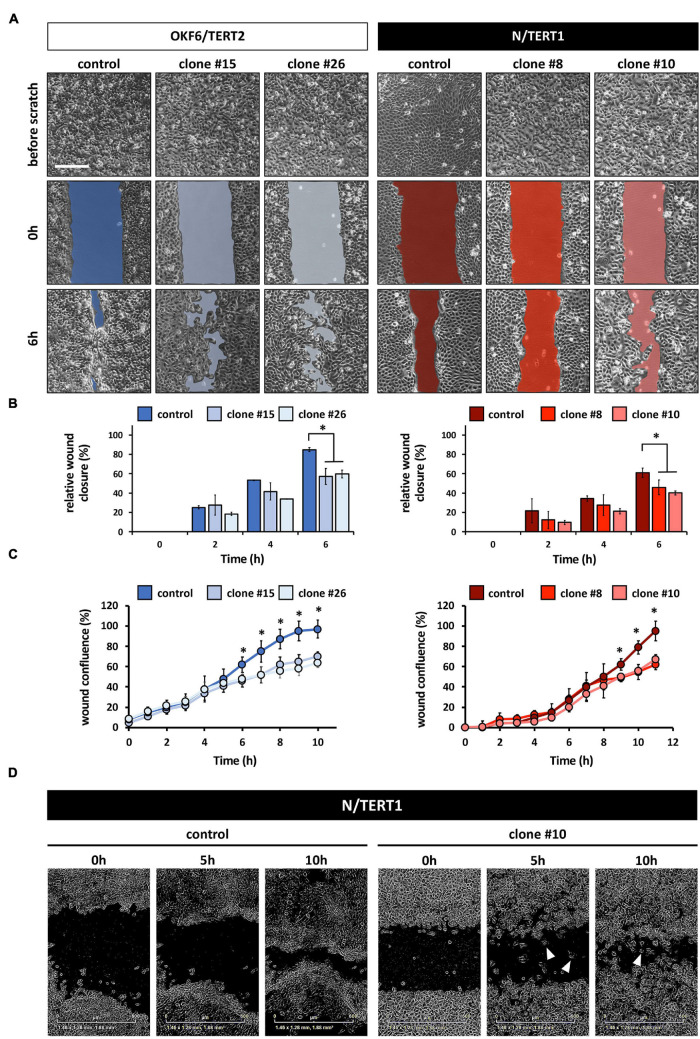
**(A)** Live Imaging pictures of the *in vitro* scratch at the indicated times after wounding the confluent monolayer and before scratching. Left side: OKF6/TERT2; Right side: N/TERT1. Scale bar: 500 μm. **(B)** Quantification of the manual scratch shows a delay of the wound closure in the absence of IRF6. ^∗^*p* < 0.05 control vs. clones. **(C)** Quantification of an automated live imaging scratch assay confirms impaired closure of the scratch in the IRF6 knockout keratinocytes compared to control. ^∗^*p* < 0.05 control vs. clones. **(D)** Pictures of N/TERT1 control and N/TERT1 clone #10 taken at the time of scratching (0 h), 5 h, and 10 h shows that lack of IRF6 results in significantly more cells that move randomly as single cells (arrowheads, right side) compared to control (left side).

Irf6 has been described as a transcription factor regulating wound healing and keratinocyte migration in mice (Biggs et al., 2014). Therefore, we wanted to assess IRF6 function in regulating migration of human postnatal keratinocytes. Lack of IRF6 in both oral mucosa- and skin-derived keratinocytes delayed the closing of an *in vitro* scratch (Figures 4A,B). These observations were confirmed by live imaging using the Incucyte Scratch Wound Assay^®^ and its automated analysis (Figure 4C). These analyses allowed us to realize that next to a delay in wound closure, the IRF6-deficient keratinocytes also differed in their pattern of migration compared to their respective controls. While the keratinocytes of both control cell lines, OKF6 and N, consistently moved as continuous epithelial sheets in a directed migration pattern, with leading edge keratinocyte in front of follower cells, IRF6-deficient keratinocytes moved preferentially as single cells, with random and undirected migration paths. Therefore, increased presence of single cells in the scratch compared to controls was a prominent hallmark of IRF6-deficient keratinocytes (Figure 4D, arrowheads). This observation is reminiscent of the morphological differences observed earlier (Figures 2A,B).

### IRF6 Is Required for the Expression and Induction of Early and Late Differentiation Markers

Since IRF6 is a master regulator of the balance between keratinocyte proliferation and differentiation (Richardson et al., 2006), we wished to analyze the proliferation rate of human postnatal keratinocytes in the absence of IRF6. Cell growth as well as qPCR analyses for the proliferation markers Proliferating-Cell-Nuclear-Antigen (*PCNA*) and *Ki67* at both LD and HD cultures did not disclose any significant differences between IRF6 knockout keratinocytes and their controls (Supplementary Figure 5).

**FIGURE 5:**
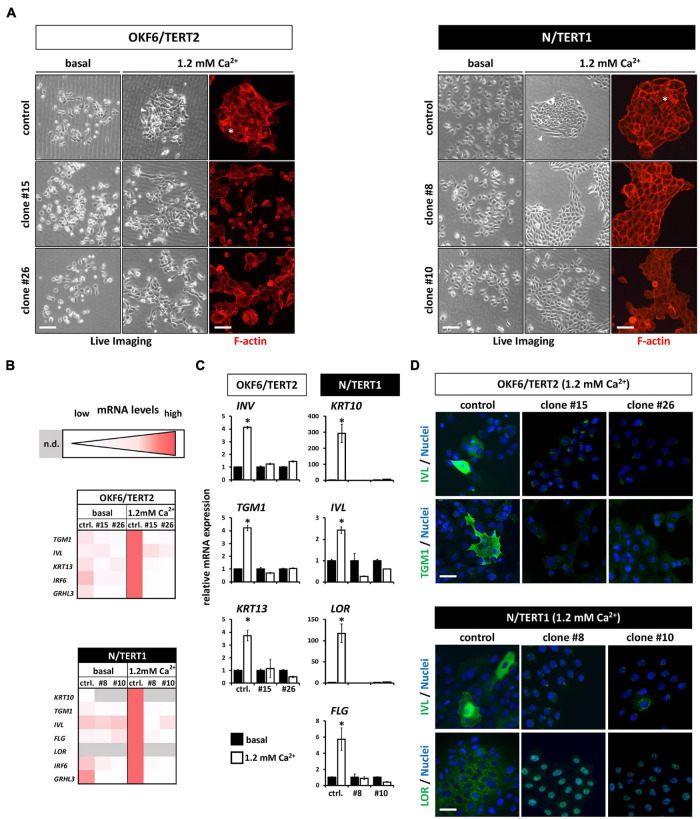
**(A)** Live Imaging pictures and F-actin staining (phalloidin, red) with or without (only Live Imaging) exogenous Ca^2+^ addition. While the control keratinocytes start to differentiate (asterisk and arrowheads), lack of IRF6 impairs differentiation. Scale bar: 200 μm (Live Imaging); Scale bar: 150 μm (F-actin). **(B)** Heatmaps of the qPCR analyses of various differentiation markers in basal (0.1 mM) vs. high Ca^2+^ (1.2 mM) conditions. Note that in the absence of IRF6 all the differentiation markers are not induced upon the Ca^2+^-switch. n.d.: not detectable (Ct > 32); ctrl.: control. **(C)** qPCR analyses of specific differentiation markers showing the lack of induction upon the addition of Ca^2+^ in the IRF6 knockout clones compared to controls. ^∗^*p* < 0.05 basal vs. 1.2 mM Ca^2+^. ctrl.: control. **(D)** Immunofluorescent staining for the markers Involucrin (IVL, green) and Transglutaminase 1 (TGM1, green) in OKF6/TERT2 cells and for IVL (green) and Loricrin (LOR, green) in N/TERT1 keratinocytes. Note that all differentiation markers were robustly induced in the control cells in the presence of IRF6. Scale bar: 50 μm. DAPI was used to counterstain the cell nuclei (blue).

Next, we assessed the differentiation potential of human keratinocytes in the absence of IRF6. Addition of exogenous Ca^2+^ to keratinocytes grown in basal growth medium induced differentiation after three days in both OKF6 and N control keratinocytes. They started to form dense colonies with signs of stratification in the center (Figure 5A, asterisks), and elongated cells at the margins (Figure 5A, arrowhead). In contrast, the IRF6-deficient keratinocytes, while also changing their morphology in response to Ca^2+^, did not show any signs of differentiation as described before (Figure 5A). To detect changes in the transcriptome in non-confluent cultures after the Ca^2+^-switch, we performed qPCR analyses for a panel of typical differentiation markers of skin- and mucosa-derived keratinocytes (Figure 5B). The Ca^2+^-switch triggered a robust induction of the early and late differentiation markers Transglutaminase 1 (*TGM1*), Involucrin (*IVL*), Keratin13 (*KRT13*), *IRF6*, and *GRHL3* in OKF6 control keratinocytes, and of *KRT10*, *TGM1*, *IVL*, Filaggrin (*FLG*), Loricrin (*LOR*), *IRF6*, and *GRHL3* in N control keratinocytes (Figures 5B,C). In contrast, IRF6-ablated keratinocytes showed an impaired differentiation capacity as none of these markers were induced in response to Ca^2+^ (Figures 5B,C). Notably, levels of some of these markers, such as *TGM1*, were already diminished in the knockout keratinocytes when compared to controls in the basal conditions, suggesting an important role of IRF6 for their regulation (Figure 5B). These results were further confirmed at protein level by staining OKF6 for IVL and TGM1, and N for IVL and LOR in controls and their corresponding IRF6 knockout clones at 1.2 mM Ca^2+^ (Figure 5D).

In addition, we subjected the IRF6 knockout keratinocytes to another, cell density-dependent *in vitro* differentiation assay (Poumay and Pittelkow, 1995). Specifically, we analyzed and compared both the cell morphology and the levels of differentiation markers in LD and HD cultures. At HD, both control cultures differentiated with enlarged cells emerging on top of the monolayer (Supplementary Figure 6A, arrowheads), while differentiation was impaired in the absence of IRF6. Analyses of various differentiation markers by qPCR, staining, and immunoblotting confirmed severe differentiation defects in IRF6-deficient keratinocytes (Supplementary Figures 6B-E). Impaired differentiation at HD in the absence of IRF6 could be at least partially rescued by ectopic expression of IRF6 and GRHL3, as assessed by increased levels of IVL and TGM1 (Supplementary Figure 7). Low levels of exogenous IRF6 in clone #8 (N cells) (Figure 2C) were not able to induce IVL and TGM1 expression, suggesting that a certain IRF6 threshold is required for its proper function.

**FIGURE 6:**
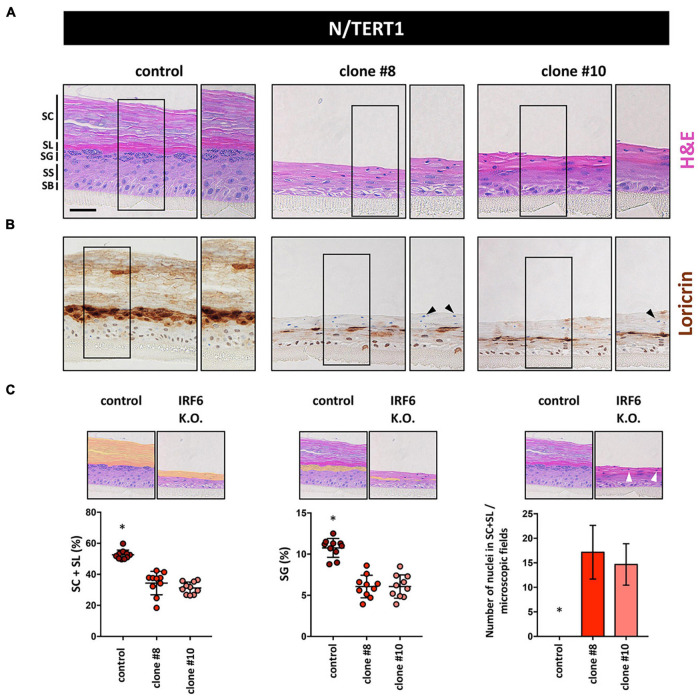
**(A)** H&E staining of 3D skin organotypic cultures obtained with N/TERT1 control and N/TERT1 clones #8 and #10 keratinocytes. The various skin layers are indicated. SB: stratum basale; SS: stratum spinosum; SG: stratum granulosum; SL: stratum lucidum; SC: stratum corneum. Scale bar: 100 μm. **(B)** Loricrin is strongly expressed in organotypic cultures of control keratinocytes in the stratum granulosum. In contrast, Loricrin is only weakly and irregularly expressed in the absence of IRF6. Close-up images are shown to the right of each organotypic culture. Arrowheads indicate aberrant presence of nuclei in the SC. **(C)** Quantification of H&E pictures of the 3D skin models. Note that in the absence of IRF6 the area of the SL + SC (orange area left panel), as well as the SG (orange area middle panel) are significantly reduced. Also, absence of IRF6 results in the presence of cell nuclei (arrowhead) in the SL and SC, which is not the case for control (right panel). ^∗^*p* < 0.05 control vs. clones #8 and #10.

These differentiation defects in 2D inspired us to use N keratinocytes for the establishment of 3D organotypic skin cultures in the absence or presence of IRF6. H&E staining of such 3D cultures revealed that while control keratinocytes were able to fully differentiate with the appearance of the typical cell layers, IRF6-deficient keratinocytes failed to build a healthy epidermis (Figure 6A). Although N keratinocytes were able to form a stratum granulosum (SG) and signs of a stratum corneum (SC) even in the absence of IRF6, these two cell layers were clearly not properly developed in IRF6-deficient cells compared to control cells. Immunohistochemical staining for the late differentiation marker LOR confirmed these observations as it was strongly and specifically expressed in the SG in control organotypic cultures. Strikingly, patchy areas of weakly LOR-positive cells were observed in the absence of IRF6 (Figure 6B). We quantified the morphological defects and measured a significantly diminished area of the two outermost layers (stratum lucidum (SL) and SC) as well as a reduced area with granulated, LOR-positive keratinocytes in the SG (Figure 6C, left and middle) in the absence of IRF6. Additionally, we observed the presence of cell nuclei in the two outermost layers when IRF6 was lacking, which is in stark contrast to controls (Figure 6C, right, arrowheads) and further confirms lack of a properly cornified layer on top of the IRF6-deficient epidermis in 3D-skin models.

### Proteomics Reveal Skin Homeostasis, Immune Response, and ECM as Major Protein Clusters Affected by the Lack of IRF6

**FIGURE 7:**
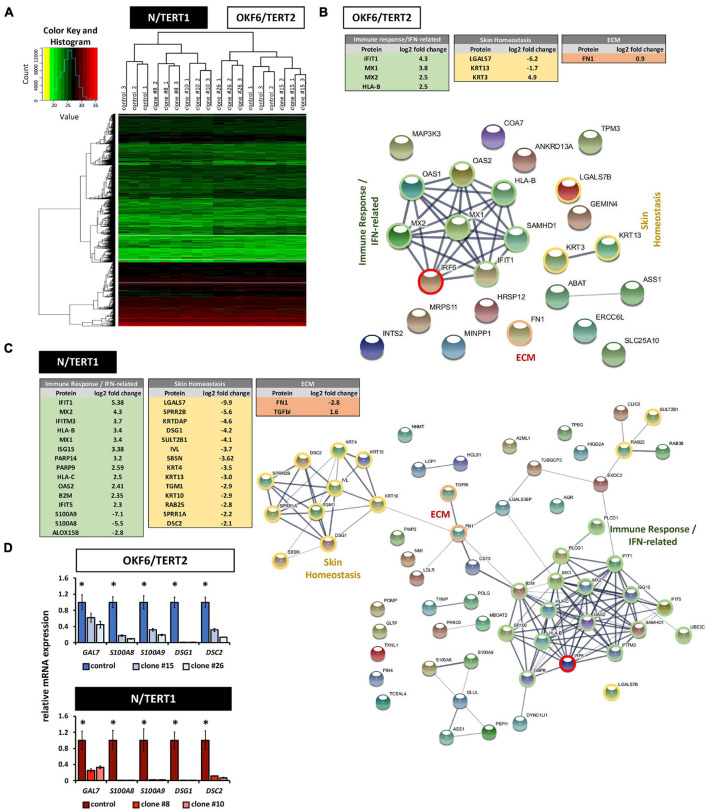
**(A)** Heat map of hierarchical clustering after imputation and color key represent the total amount of identified proteins in N/TERT1 (left side) and OKF6/TERT2 samples (right side). Note the hierarchical clusters on top demonstrating significant changes in the proteome between skin- and oral mucosa-derived cell lines. **(B)** Differentially expressed proteins in OKF6 clone #26 vs. OKF6 control in cultures at high cell density (HD). Tables on the top summarize the main three protein clusters: immune response/IFN-related (green), Skin Homeostasis (yellow), and ECM (red). String network depicting all differentially expressed proteins is shown as well with the three main clusters as described before. Note that only proteins are shown having a differential expression of at least log_2_ fold change = 1. ECM: extracellular matrix. **(C)** Differentially expressed proteins in N clone #10 vs. N control in cultures at high cell density (HD). Tables on the left summarize the main three protein clusters: immune response/IFN-related (green), Skin Homeostasis (yellow), and ECM (red). String network depicting all differentially expressed proteins is shown as well with the three main clusters as described before. Note that only proteins are shown having a differential expression of at least log_2_ fold change = 1. ECM: extracellular matrix. **(D)** qPCR verifications for some mRNA transcripts (*GAL7*, *S100A8*, *S100A9*, *DSG1*, and *DSC2*) for which the corresponding proteins have been found to be differentially expressed in both OKF6 and N cells compared to the corresponding IRF6-deficient keratinocytes. ^∗^*p* < 0.05 controls vs. clones #15 and #26, and clones #8 and #10.

To learn more about IRF6 function in both skin and oral mucosa, the proteomes of IRF6-deficient OKF6 and N cells were analyzed and compared to their corresponding controls. A total of roughly 3,200 proteins were identified (Supplementary Data Sheet 2 and Supplementary Figure 8). Normalized heat maps as well as hierarchical clustering revealed different proteomic profiles between OKF6/TERT2 and N/TERT1, and between their IRF6 knockout clones (Figure 7A). Employing strict criteria for the analysis (log_2_ fold change >1 (except for FN, which we confirmed by immunoblotting), we determined roughly 70 differentially expressed proteins in N control compared to IRF6 knockout clones (Supplementary Table 3) and only about 20 proteins for OKF6 (Supplementary Table 4). A selection of differentially expressed proteins could be categorized to the main functional groups “Skin Homeostasis,” “Immune response/IFN-related”, and “ECM” in both the cell lines (Figure 7B,C). To further identify protein clusters among the differentially expressed proteins and their putative biological functions, these proteins were inserted into the STRING database for potential networks. STRING networks were retrieved indicating the main protein clusters affected by the lack of IRF6 (Figures 7B,C): Immune response/IFN-related (green), Skin Homeostasis (yellow), and ECM (red). We verified a panel of the identified proteins by analyzing their transcript levels. qPCR analyses for the genes Galectin-7 (*GAL7*), *S100A8*, *S100A9*, Desmoglein-1 (*DSG1*), and Desmocollin-2 (*DSC2*) showed that lack of IRF6 significantly decreased all their transcript levels (Figure 7D). In conclusion, our data show that IRF6 plays an important role in the regulation of skin homeostasis and keratinocyte-effected immune response in both oral mucosa- and skin-derived postnatal human keratinocytes.

## Discussion

Most of our knowledge on IRF6 has been gained from animal models and murine cells. Yet, studying IRF6 in human cells is important since *IRF6* variants are associated with non-syndromic CLP cases as well as causative for two syndromic forms of orofacial clefting, VWS and PPS (Kondo et al., 2002). In addition, *IRF6* has been found mutated in head and neck squamous cell carcinoma (Stransky et al., 2011) and IRF6 is often lost or downregulated in many solid cancers (Bailey et al., 2008; Botti et al., 2011). Still, complete and detailed knowledge on IRF6 function, its upstream and downstream effectors in human postnatal keratinocytes remains elusive. Our study on IRF6 function in postnatal human keratinocytes mostly confirms and complements the various studies using embryonic murine *Irf6^–/–^* keratinocytes (Biggs et al., 2012, 2014; Rhea et al., 2020). The two most apparent defects in *IRF6*-ablated keratinocytes were phenotypes related to cell morphology and to epidermal homeostasis.

We applied two distinct *in vitro* differentiation assays to assess the differentiation potential of human skin- and oral mucosa-derived keratinocytes in the absence of IRF6. In both cell lines, disruption of IRF6 impaired the entire epidermal differentiation program. The early (e.g., *KRT10*) as well as late (e.g., *LOR*) differentiation markers were deregulated in IRF6-deficient keratinocytes compared to their controls under differentiating conditions (Figure 5 and Supplementary Figure 6). We also noted some variations between the two *in vitro* differentiation assays. While the Ca^2+^-switch did not induce any of the differentiation markers in both cell lines in the absence of IRF6, the cell density-dependent differentiation triggered an induction of some of the markers, whose levels however remained significantly lower than in the control. We conclude that IRF6 is important for regulating early as well as late differentiation events in both oral mucosa and skin. In agreement with this observation, 3D organotypic skin models indicated that IRF6 knockout keratinocytes failed to build a healthy and regular epidermis. The SG as well as the SC appeared inadequately formed in the absence of IRF6, as both were significantly thinner compared to the ones built in controls. Furthermore, the SG did not develop properly, but rather patchy, which was confirmed by staining for LOR (Figures 6B,C). LOR levels were significantly decreased compared to control, and only a small fraction of keratinocytes displayed a highly heterogenous positivity. Also, abnormal presence of nucleated cells in the stratum corneum, a phenotype originally described as parakeratosis (Cardoso et al., 2017), was often observed in the absence of IRF6. Similar traits have been reported in receptor-interacting protein kinase 4 (*Ripk4*)-deficient mice. Such mice exhibit patchy areas of LOR-positive corneocytes and nucleated cells in the outermost epidermal layer (De Groote et al., 2015), highlighting a role for the described RIPK4-IRF6 connection in epidermal integrity (Kwa et al., 2015; Oberbeck et al., 2019). Strikingly, *Irf6* null embryos display an expanded spinous layer and complete lack of the stratum granulosum and corneum (Ingraham et al., 2006). Similarly, embryonic murine *Irf6*^–/–^ keratinocytes are not competent to undergo terminal differentiation *in vitro*, but Irf6 was not necessary for Krt10 induction (Biggs et al., 2012). In contrast, our data reveal that IRF6 is already essential for *KRT10* induction and that an irregular, probable dysfunctional SC is still formed in the absence of IRF6. These discrepancies might be either related to differences between mice and humans or to the fact that embryonic is compared to postnatal tissue. In human, it has been shown that *IRF6*-associated VWS patients exhibit increased keratinocyte proliferation, but normal KRT10 levels when compared to non-syndromic CLP cases (Hixon et al., 2017). This suggests that even reduced IRF6 levels, as often is the case in VWS patients (Degen et al., 2020), still are sufficient to properly regulate KRT10 expression. Following this idea, we provide evidence that forced expression of *IRF6* in the IRF6-deficient keratinocytes is capable of partially rescuing the morphological and differentiation phenotypes, but only, if a certain IRF6 threshold is attained (Figure 2C and Supplementary Figure 7). Such observations have already been reported in studies using *IRF6*^–/–^ embryos (Kousa et al., 2017) and *Irf6*^+^*^/^*^–^ mice (Rhea et al., 2020). In addition, we show that GRHL3, a downstream target of IRF6, is able to partially rescue the differentiation defect in IRF6-depleted keratinocytes, which fits with the literature (de la Garza et al., 2013). However, exogenous GRHL3 was not capable of rescuing the scattered cell colony morphology (Supplementary Figure 4B). GRHL3 and IRF6 are transcription factors expressed in the periderm during embryogenesis (Richardson et al., 2009; Peyrard-Janvid et al., 2014; Richardson et al., 2014), regulate keratinocyte differentiation (Ting et al., 2005), and are associated with CLP and VWS (Kondo et al., 2002; de la Garza et al., 2013; Peyrard-Janvid et al., 2014; Mangold et al., 2016). How they work together to dictate keratinocyte differentiation is not fully elucidated yet and needs more detailed investigations. Our data suggest a common function during differentiation, but an IRF6-specific activity controlling cell morphology.

Lack of epidermal integrity in the absence of IRF6 was already visible in the stratum basale in the 3D organotypic cultures, which was irregular and disordered (Figure 6A). This observation fits to the second major phenotype in IRF6-depleted keratinocytes: The appearance of altered cell size and colony morphology. Cell colonies of IRF6-depleted cells appeared scattered (reduced colony circularity) with less stable cell-cell contacts and there was a significantly increased number of single and enlarged cells in these cultures when compared to control (Figure 2). This phenotype was more pronounced in the skin-derived keratinocytes, which might reflect our observation that IRF6 levels are higher in skin- than in oral mucosa-derived keratinocytes (Figure 1). Single cells trying to evade the stable cell colonies and increased cell size are features associated with an EMT (Lamouille and Derynck, 2007). Although IRF6-deficient keratinocytes displayed slightly increased levels of some mesenchymal markers (e.g., Fibronectin), E-Cadherin levels remained unchanged, and the growth factor-triggered EMT was less pronounced when compared to controls (Figure 3). In addition, EMT induction by TGFβ1 resulted in increased levels of *IRF6*, which is identical to the response of Irf6 to TGFβ3 in mice (Ke et al., 2015). This observation might be unexpected as IRF6 is described as an epithelial marker. However, it is clear that IRF6 is not a strict epithelial marker as it is also expressed in immune cells (Joly et al., 2016) and involved in osteoblast differentiation of craniofacial bone (Thompson et al., 2019). Furthermore, it has been shown that ectopic Irf6 expression enhances *Snai2* mRNA levels in mice (Ke et al., 2015), which consequently leads to the repression of *Cdh1*. A very recent study reports that IRF6 is responsible for the regulation of E-Cadherin to the plasma membrane, which is important for the maintenance of cohesion between epithelial cells (Antiguas et al., 2021). All these data imply that EMT is partially dependent on IRF6, which is in agreement with a previous *in vivo* study in mice (Ke et al., 2015), and that epithelial cell characteristics are maintained, even in the absence of the epithelial-specific transcription factor IRF6.

Further support in this regard was obtained from the observation that the closure of an *in vitro* scratch was delayed in the absence of IRF6 (Figure 4). Such a result is not conform with a more detached migratory state of mesenchymal cells. Notably, IRF6-depleted keratinocytes not only closed the wound slower, but they also moved in a different migration pattern when compared to control. Instead of moving as a continuous epithelial cell sheet to close the artificial wound like control keratinocytes, IRF6-deficient cells preferentially moved as single cells. This observation is reminiscent of the morphological phenotypes described above and complements previous studies using embryonic *Irf6^–/–^* keratinocytes showing reduced migration speed and less directionality (Biggs et al., 2014). Whether this irregular migration pattern *in vitro* provides the molecular rationale for the observed wound healing complications observed in VWS patients *in vivo* remains to be elucidated and needs additional investigations. Our data point toward a crucial role for IRF6 in regulating intrinsic postnatal cellular characteristics and processes. However, cell growth was not affected by IRF6 in both OKF6 and N keratinocytes (Supplementary Figure 5). Although similar results were obtained in mice, *Irf6* knockout attenuated the long-term proliferative potential of the cells (Biggs et al., 2012). We refrained from such long-term experiments, since we used TERT immortalized cells potentially masking any effect on cell proliferation by the absence IRF6.

Proteomics of differentiated keratinocytes confirmed our *in vitro* differentiation data. Most of the differentiation markers (e.g., *KRT10*, *TGM1*, *KRT13*, *IVL*) were downregulated in the absence of IRF6 in both cell lines compared to control (Figure 7). Unfortunately, LOR and FLG were not detected in our proteomic analysis for technical reasons. Several candidate proteins that might explain the morphological phenotypes observed in IRF6-deficient keratinocytes were identified as well. Among them were Desmocollin-2 (DSC2), Desmoglein-1 (DSG1), and Galectin-7 (LGALS7), which were all significantly reduced in the absence of IRF6 (Figure 7). DSC2 and DSG1 are components of desmosomes (Garrod and Chidgey, 2008), which represent intercellular junctions essential for mediating cohesion, epidermal integrity, and MAPK/ERK signaling regulation (Green et al., 2010; Harmon et al., 2013). Disruption of desmosomes has been attributed with loss of cell-cell contacts, EMT, impaired skin differentiation, and lack of epidermal barrier formation (Johnson et al., 2014). Whether IRF6-deficient keratinocytes completely lose their desmosomes or whether only certain of their components are altered remains to be discovered. It is worth mentioning that in the *Irf6*^–/–^ mice, desmosomes were observed throughout the epidermis including the most superficial keratinocyte layers in contrast to wt animals lacking desmosomes in the superficial layers (Ingraham et al., 2006). Another study confirmed that *Irf6* knockout murine keratinocytes did not affect the regulation of the components of desmosomes (Ferone et al., 2013). Two proteins, RAB25 and SULT2B2, which are known to be associated with healthy skin and the regulation of epidermal differentiation and proliferation, have also been found to be robustly decreased in the absence of IRF6 in skin-derived keratinocytes (Heinz et al., 2017; Jeong et al., 2019).

LGALS7 is a protein belonging to a family of soluble lectins and shows preferential expression in stratified epithelia, including the skin and oral cavity (Magnaldo et al., 1998), where it has crucial functions for skin homeostasis in response to major challenges, and for the regulation of keratinocyte proliferation and differentiation (Gendronneau et al., 2008; Chen et al., 2016). Strikingly, LGALS7 also controls re-epithelialization of wounds by regulating directionality, collective cell behavior, and intercellular E-Cadherin-dependent adhesions (Cao et al., 2003; Advedissian et al., 2017). Proteomics also identified up-regulation of the extracellular matrix protein Fibronectin (FN), which is involved in various cellular aspects, for instance wound healing (Pankov and Yamada, 2002). Nothing is known yet about an IRF6-FN connection, which might suggest an important role for IRF6 in epithelial-mesenchymal interactions and might explain the maturation defect of the granulation tissue observed during wound healing in heterozygous *Irf6*^+^*^/^*^–^ mice (Rhea et al., 2020). In general, proteomics exposed more changes upon IRF6 depletion in the skin-derived N/TERT1 keratinocytes compared to the oral mucosal OKF6/TERT2. This might reflect our observations that IRF6 is higher expressed in skin than in oral mucosa (Figure 1), a tissue that lacks the outermost cornified skin layers.

Skin integrity is achieved by formation of a properly built skin barrier and by the adequate communication with immune cells, including the release of cytokines and chemokines. Both processes protect us from various external insults and guarantee the maintenance of physiological skin homeostasis. Using human postnatal keratinocytes we demonstrate that both crucial functions of the skin are affected by the absence of IRF6. Our experimental data reveal major functions for IRF6 in the differentiation program as well as in the regulation of cell-cell contacts. Moreover, we also show that many “immune response/IFN-related” proteins are altered in the absence of IRF6. While a lot of knowledge has been gained in the recent years about the function of IRF6 during various aspects of craniofacial development, a complete understanding of the transcription factor is still missing. Especially in postnatal human tissues, knowledge about IRF6 remains sparse. One limitation of our study is the use of immortalized keratinocytes for elucidating the role of IRF6 in human cells. While all our data using two cell lines are in line with the literature on Irf6 function in mice, more investigations are warranted to better understand IRF6 in primary human cells and tissues.

## Data Availability Statement

The mass spectrometry proteomics data have been deposited to the ProteomeXchange Consortium via the PRIDE partner repository with the dataset identifier PXD027669. Most of the data is reported in the Supplementary Files section.

## Ethics Statement

This work was performed according to the Ethical Principles for Medical Research Involving Human Subjects as defined by the World Medical Association (Helsinki declaration)^7^. Isolation of foreskin-, and oral mucosa-derived keratinocytes for this study has been approved by the Kantonale Ethikkommission of Bern, Switzerland (protocol number: 2017-01394). Written consent was obtained from the parents of the children.

## Author Contributions

EG, LM, LP, SR, and MD performed the experiments and analyzed the data. SvG, CK, and MD planned, coordinated and designed the experiments, and the project. EG and MD wrote the manuscript. SvG and CK provided support throughout the project. All authors critically reviewed the manuscript.

## Conflict of Interest

The authors declare that the research was conducted in the absence of any commercial or financial relationships that could be construed as a potential conflict of interest.

## Publisher’s Note

All claims expressed in this article are solely those of the authors and do not necessarily represent those of their affiliated organizations, or those of the publisher, the editors and the reviewers. Any product that may be evaluated in this article, or claim that may be made by its manufacturer, is not guaranteed or endorsed by the publisher.

## Footnotes

1
https://imagej.nih.gov/ij/

2
www.pantherdb.org

3
www.string-db.org

4
http://jvenn.toulouse.inra.fr./app/index.html

5
www.ncbi.nlm.nih.gov/tools/primer-blast

6
www.cse-lab.ethz.ch/software/

7
https://www.wma.net/policies-post/wma-declaration-of-helsinki-ethical-principles-for-medical-research-involving-human-subjects
