# Supplementary material for: Lack of IRF6 Disrupts Human Epithelial Homeostasis by Altering Colony Morphology, Migration Pattern, and Differentiation Potential of Keratinocytes
Source: Front Cell Dev Biol. 2021 Sep 30;9:718066. doi: 10.3389/fcell.2021.718066 (PMC8514984; doi:10.3389/fcell.2021.718066)
Supplement: Supplementary file 1 [file Data_Sheet_1.pdf]

Supplementary Material

Lack of IRF6 disrupts human epithelial homeostasis by altering colony morphology, migration pattern, and differentiation potential of keratinocytes

Eleftheria Girousi [1], Lukas Muerner [2], Ludovica Parisi [1], Silvia Rihs [1], Stephan von Gunten [2], Christos Katsaros [1] and Martin Degen [1,*]

*** Correspondence:** Martin Degen
martin.degen@zmk.unibe.ch

1 Supplementary Figures and Tables

1.1. Supplementary Figures

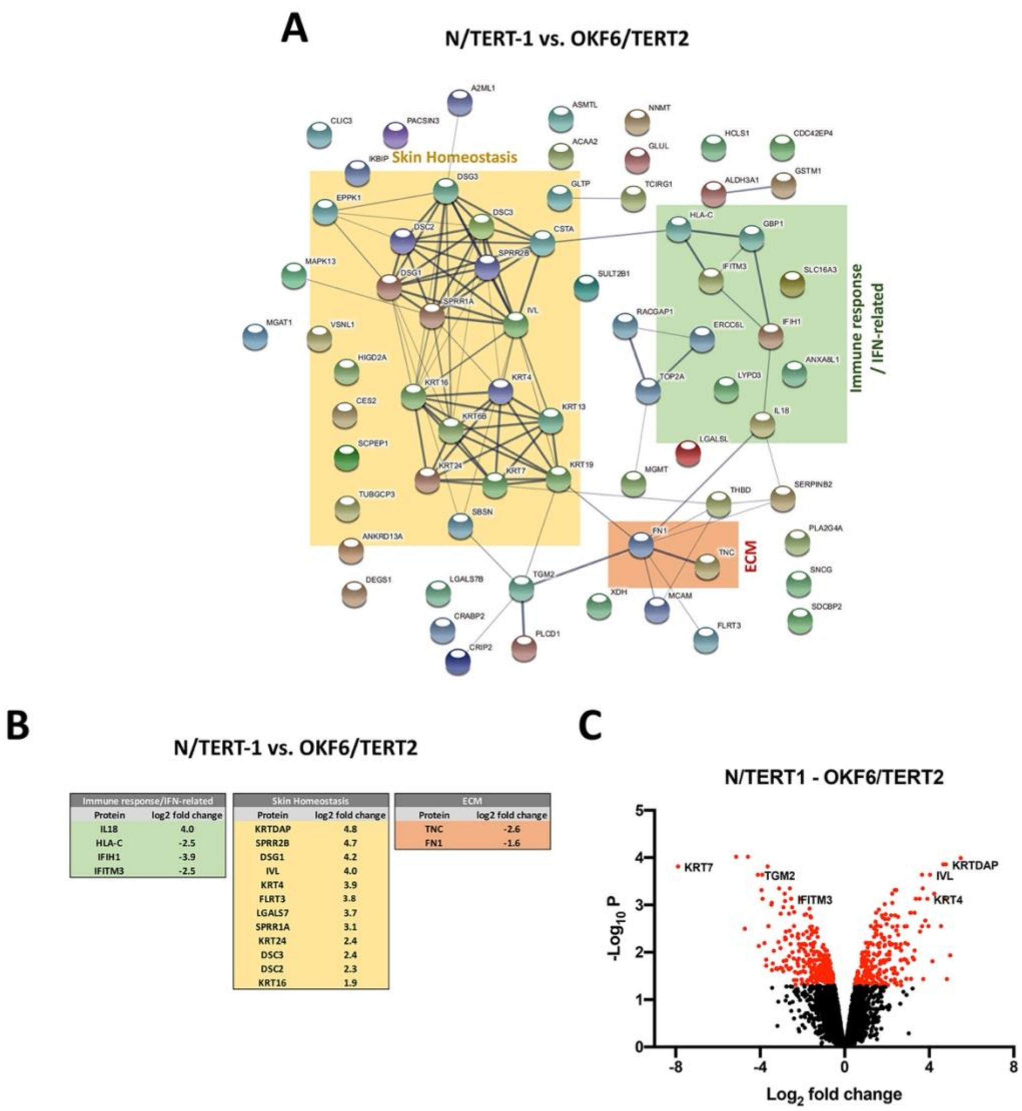

Supplementary Figure 1. (A) Proteomic analysis of OKF6/TERT2 vs. N/TERT1 keratinocytes at high cell-density. Differentially expressed proteins are shown in protein-protein interaction networks using the String database. Yellow: protein cluster "Skin Homeostasis"; Green: protein cluster "immune response / IFN-related"; Red: protein cluster "Extracellular Matrix" (ECM). **(B)** List of selected proteins including their log2 fold change belonging to the three main protein clusters. **(C)** Volcano plot of the proteomic analysis. The red dots represent the significantly differentially expressed proteins ($-Log_{10}$ p >1.3).

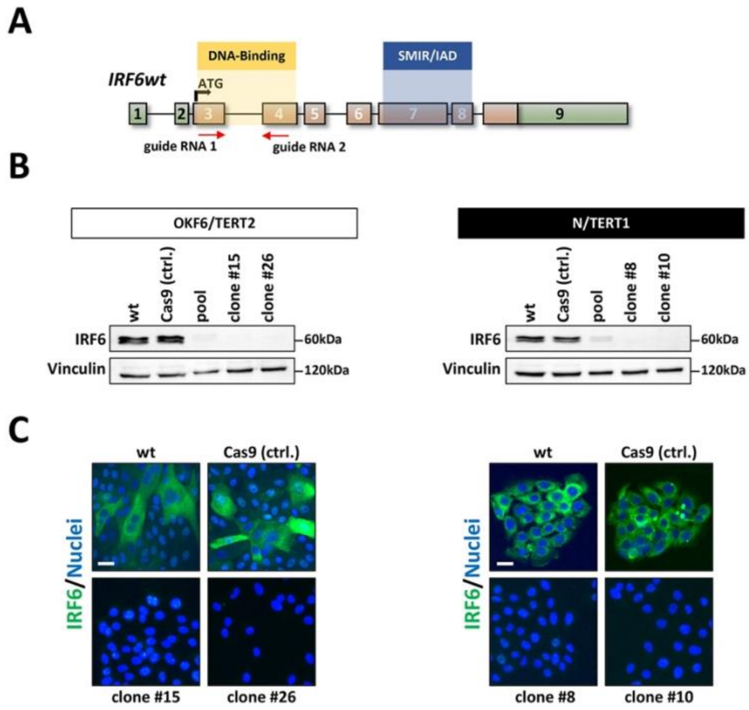

Supplementary Figure 2. (A) Genomic structure of the *IRF6* gene and the position of the IRF6-specific guide RNAs targeting exon 3 and exon 4, which encode for the DNA-binding domain. Green boxes: untranslated exons; Orange boxes: translated exons; SMIR/IAD: SMAD/IRF (SMIR)/interferon association domain (IAD). **(B)** Confirmation of the IRF6 knockout by immunoblots. The two single cell-derived OKF6/TERT2 clones #15 and #26 (left) and the two single cell-derived N/TERT1 clones #8 and #10 (right) do no longer express detectable amounts of IRF6 compared to IRF6 levels in their respective parental and Cas9-expressing (control) cell lines as well as in the bulk of the cells (pool) after CRISPR/Cas9. Note that the cell pools still show some low levels of IRF6 and that the Cas9-expressing cells are identical to the parental ones. kDa: kilo Dalton; wt: wildtype; ctrl.: control. **(C)** Immunofluorescent analysis of the IRF6 knockout clones using an anti-IRF6 antibody reveals and confirms absence of IRF6. Note that endogenous IRF6 is mainly expressed in the cytoplasm. Left side: OKF6/TERT2; Right side: N/TERT1. Scale bars: 50 µm; ctrl.: control. Full-length immunoblots are shown in **Supplementary Figure 9**.

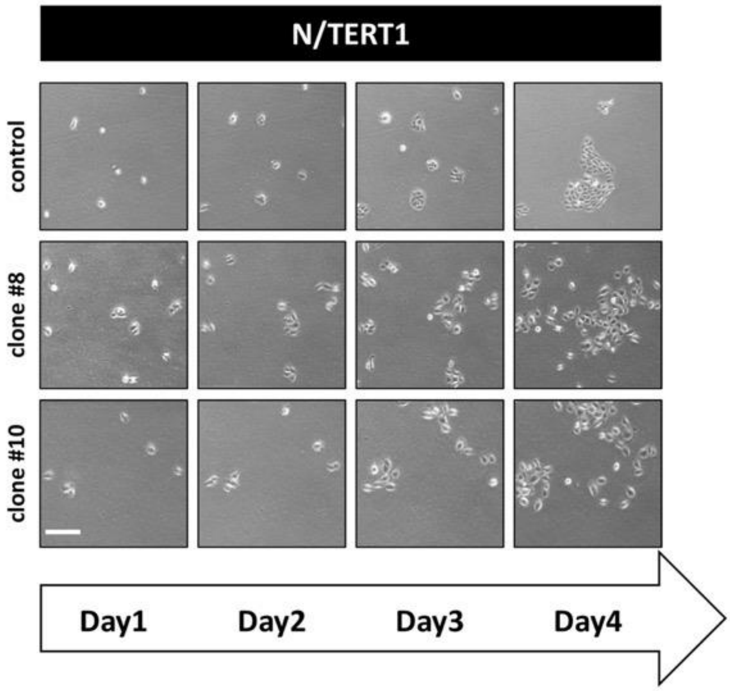

Supplementary Figure 3. N/TERT1 control as well as both cell clones of the IRF6 knockout were seeded at low density and every day a picture of the same spot was taken. Note the formation of cohesive colonies in the control while the cells remain scattered in the absence of IRF6.

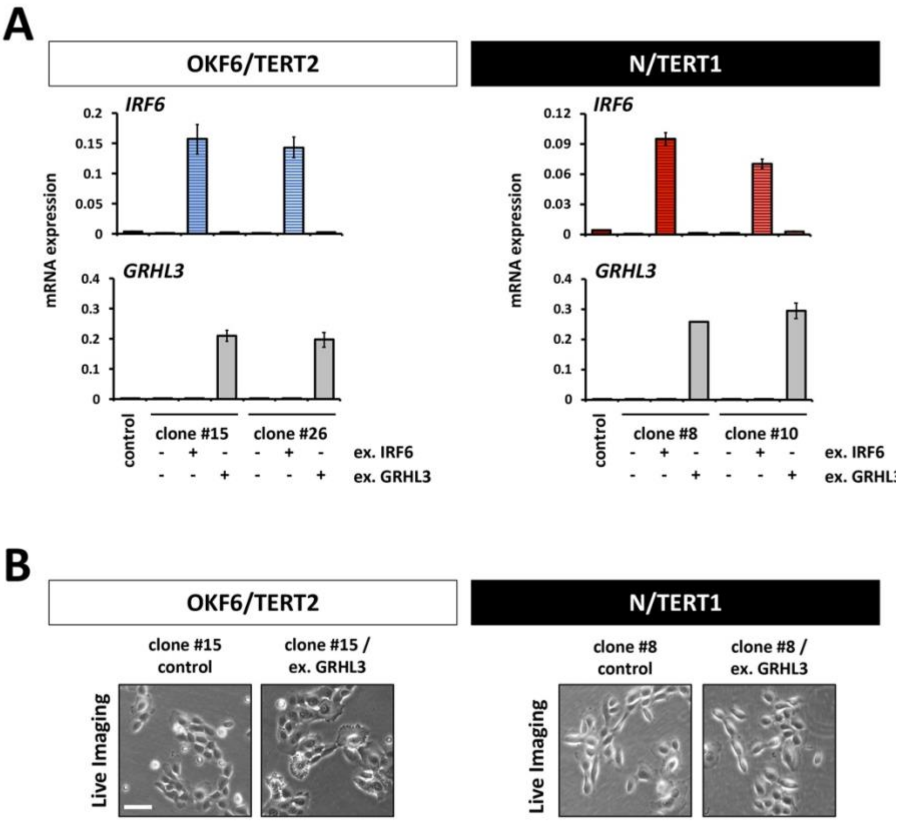

Supplementary Figure 4. (A) Confirmation of the IRF6 and GRHL3 over-expression in IRF6 knockout clones by qPCR. Note that in N/TERT (right side) exogenous IRF6 is constantly knocked-out, while in OKF6 (transient transfection of the guide RNA-containing plasmid) IRF6 is strongly overexpressed. ex.: exogenous; kDa: kilo Dalton. **(B)** Live imaging pictures of typical cell colonies of the control and GRHL3 over-expressing cells. Note that the morphology defects cannot be rescued by GRHL3. Scale bar: 100 µm.

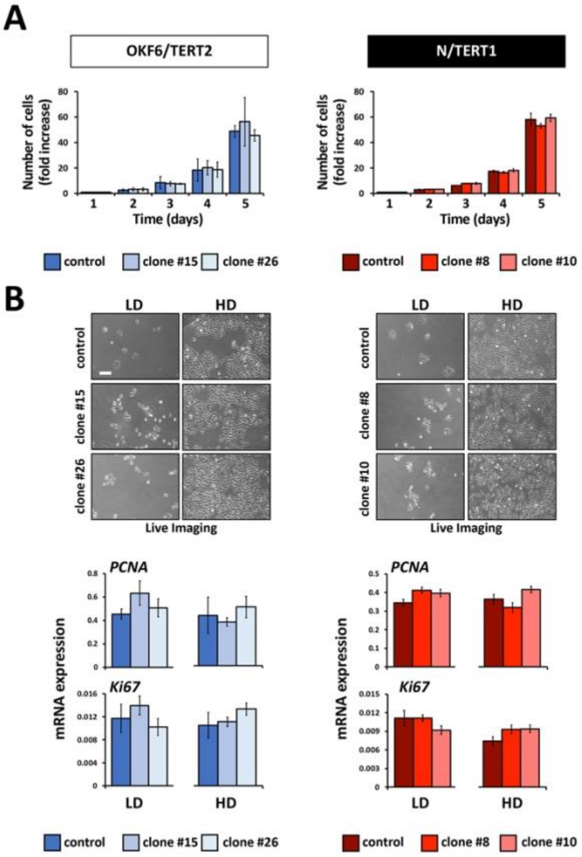

Supplementary Figure 5. (A) Cell growth analysis of control and IRF6 knockout OKF6/TERT2 (left side) and N/TERT1 keratinocytes (right side). Note that *in vitro*, absence of IRF6 does not significantly affect proliferation rate within 5 days. **(B)** Live imaging pictures and qPCR analyses of the cell cultures grown to low density (LD, left) and high density (HD, right) for the proliferation markers *PCNA* and *Ki67* do not reveal any differences in control when compared to IRF6 knockout keratinocytes. Scale bar: 500 µm.

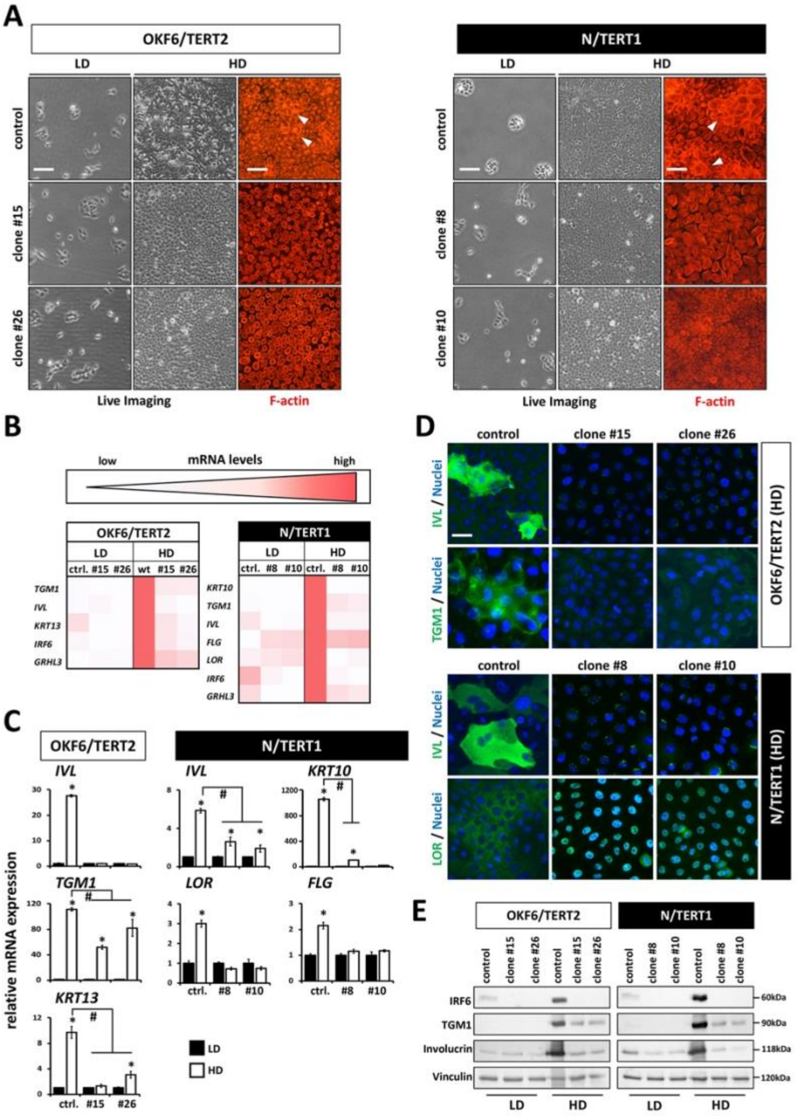

Supplementary Figure 6. (A) Live imaging pictures and F-actin staining (phalloidin, red) analyzing the cell density-driven differentiation, which is clearly visible in controls (arrowheads), but not in the IRF6 knockout clones. Scale bar: 200 µm (Live Imaging); Scale bar: 150 µm (F-actin). **(B)** Heatmap of the qPCR analyses of various differentiation markers in low density (LD) vs. high density (HD) cultures. Note the lack of induction of differentiation markers at HD in the absence of IRF6. ctrl.: control. **(C)** Graphs of the qPCR analyses of specific differentiation markers showing the lack of induction (*IVL*, *KRT13* in OKF6/TERT2 IRF6 knockout clones; *LOR*, *FLG* in N/TERT1 IRF6 knockout clones) or significantly reduced levels of differentiation markers (*TGM1* and *KRT13* in OKF6/TERT2 clones; *IVL* and *KRT10* in N/TERT1 clones) upon reaching confluence in the IRF6 knockout clones compared to controls. *$p<0.05$ HD vs. LD; # $p<0.05$ control vs. clones. ctrl.: control. **(D)** Immunofluorescent staining for the markers Involucrin (IVL, green) and Transglutaminase (TGM1, green) in OKF6/TERT2 cells and for IVL (green) and Loricrin (LOR, green) in N/TERT1 keratinocytes. Note that all differentiation markers were robustly induced in the control cells in the presence of IRF6 at HD. Scale bar: 50 µm. DAPI was used to counterstain the cell nuclei (blue). **(E)** Immunoblots for the proteins IRF6, TGM1, and Involucrin confirms defective differentiation capacity in the absence of IRF6 compared to control. kDa: kilo Dalton. Full-length immunoblots are shown in **Supplementary Figure 9**.

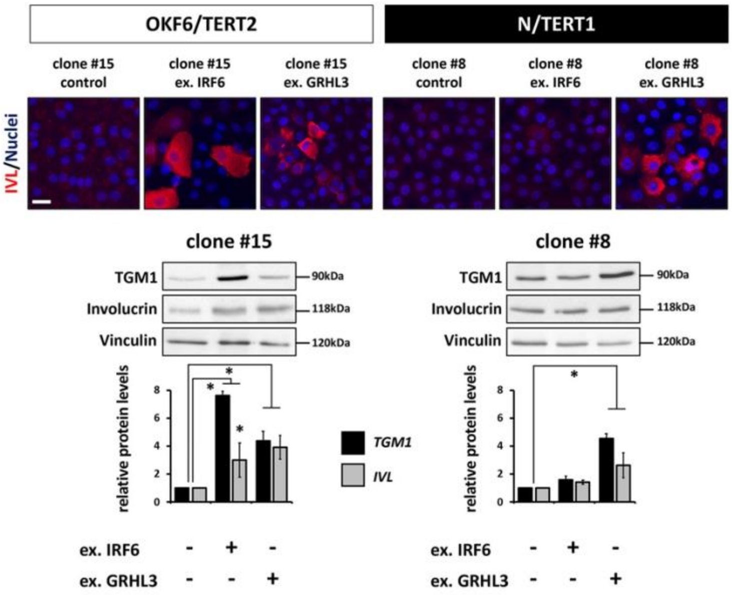

Supplementary Figure 7. Forced expression of IRF6 and GRHL3 is able to rescue the differentiation defects in IRF6-deficient keratinocytes as assessed by staining for the differentiation marker IVL and by immunoblotting for TGM1 and IVL. Quantification of the immunoblots is shown below the blots as relative protein levels. Note that in N/TERT1 keratinocytes, exogenous IRF6 is only minimally expressed and is not able to rescue the differentiation defects. Scale bar: 200 µm. *$p<0.05$. Full-length immunoblots are shown in **Supplementary Figure 9**.

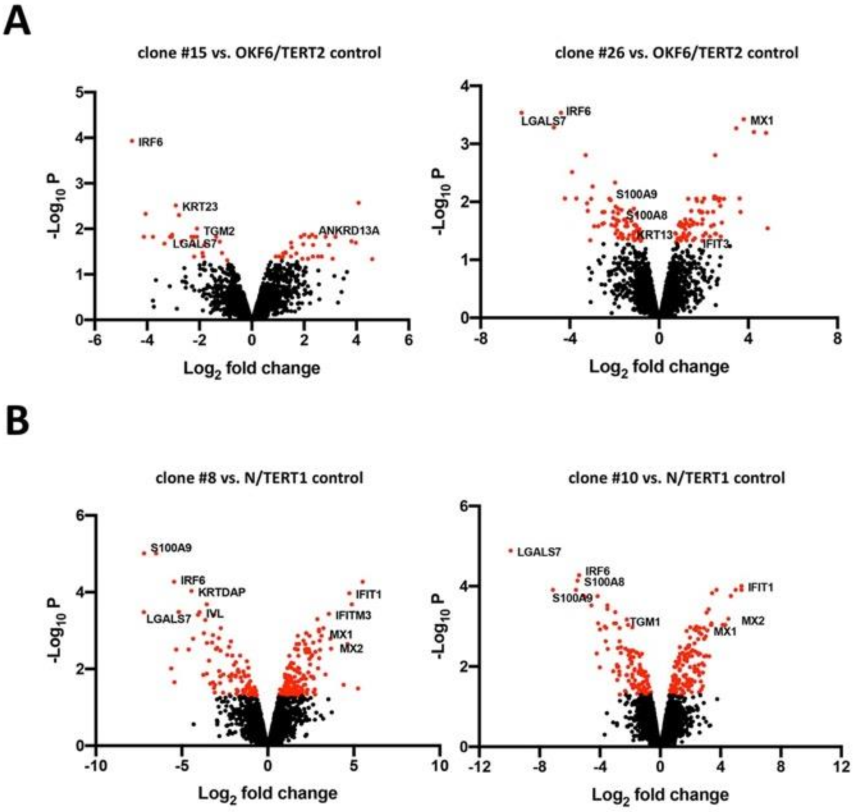

Supplementary Figure 8. (A) Volcano plots of the proteomic analysis in OKF6/TERT2 control vs. OKF6/TERT2 clone #15 and clone #26. The red dots represent the significantly differentially expressed proteins. (Log_{10} p >1.3). **(B)** Volcano plots of the proteomic analysis in N/TERT1 control vs. N/TERT1 clone #8 and clone #10. The red dots represent the significantly differentially expressed proteins. (Log_{10} p >1.3).

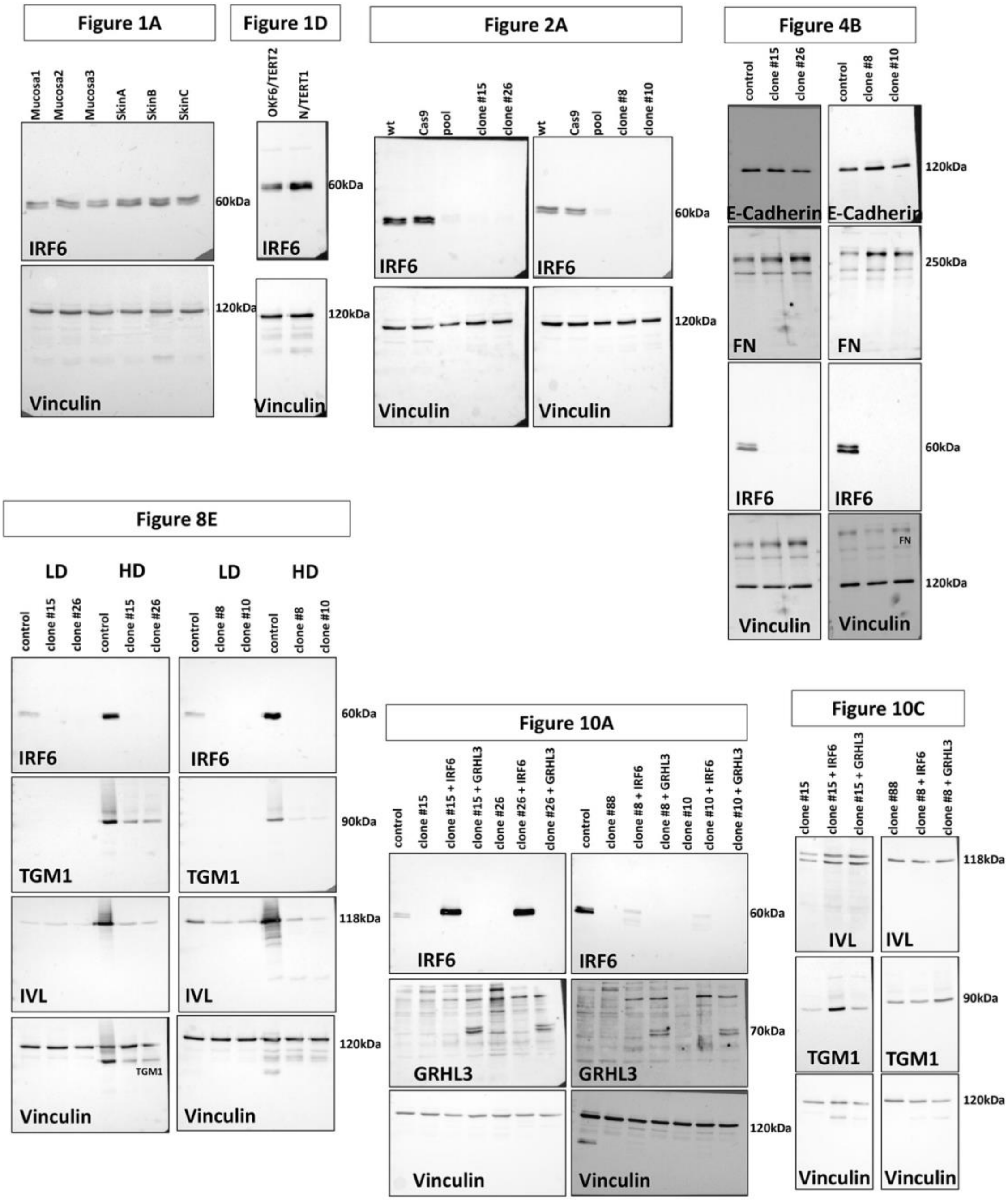

Supplementary Figure 9. Images of the full-length blots of all immunoblotting experiments. Molecular weights are indicated to the right of the blots. Detected proteins are indicated on the bottom of each blot. Note that sometime membranes were reprobed with different antibodies.

1.2. Supplementary Tables

GENE	SEQUENCE FWD	SEQUENCE REV	AMPLICON (BP)
**Interferon Regulatory Factor 6 *(IRF6)***	GCTCTTCCATATCATGGCCCTC	CTACAGCCCAGGCCTTAAAAA	200
**Grainyhead-like Regulatory Factor 3 *(GRHL3)***	CCCCCATGTCCAGAGGACTA	ACTCGCCTGACTTGATGTGG	91
**E-Cadherin 1 *(CDH1)***	AGAACGCATTGCCACATACACT	TCTGATCGGTTACCGTGATCAA	101
**Fibronectin *(FN)***	CCATTATTGGGTACCGCATCACA	AGGAGGAACAGCCGTTTGTTGT	200
**Vimentin *(VIM)***	TGTCCAAATCGATGTGGATGTTTC	TTGTACCATTCTTCTGCCTCCTG	117
**Snail1 *(SNAIL)***	AAGATGCACATCCGAAGCC	CGCAGGTTGGAGCGGTCAGC	164
**Proliferating cell nuclear antigen *(PCNA)***	CGACACCTACCGCTGCGACC	TAGCGCCAAGGTATCCGCGT	133
Ki67	TGACTTCCTTCCATTCTGAAGAC	TGGGTCTGTTATTGATGAGCC	109
**Involucrin *(IVL)***	GGCCCTCAGATCGTCTCATA	CACCCTCACCCCATTAAAGA	131
**Loricrin *(LOR)***	AGACCCAGCAGAAGCAGGCG	AGCAGAACTAGATGCAGCCG	200
**Keratin 10 *(KRT10)***	TGGTTCAATGAAAAGAGCAAGGA	GGGATTGTTTCAAGGCCAGTT	151
**Keratin 13 *(KRT13)***	CTGAACAAGGAGGTGTCTACCA	ATAGCGGCACTCCGTCTCT	162
**Transglutaminase 1 *(TGM1)***	CCCCCGCAATGAGATCTACA	ATCCTCATGGTCCACGTACACA	73
**Filaggrin *(FLG)***	CTGGACACTCAGGTTCCCAT	TTTCGTGTTTGTCTGCTTGC	103
**Galectin-7 *(GAL7)***	TTGGTTCCTCCCAATGCCAGCA	CTCCTTGCTGTTGAAGACCACC	126
S100A8	ATGCCGTCTACAGGGATGACCT	AGAATGAGGAACTCCTGGAAGTTA	142
S100A9	GCACCCAGACACCCTGAACCA	TGTGTCCAGGTCCTCCATGATG	124
**Desmoglein-1 *(DSG1)***	CTGCTGGCATTGGACTCCTCAT	CATTCGGGAACAGGCTCAAAGC	124
**Desmocollin-2 *(DSC2)***	CACAGAAGCTCCTGGAGATGAC	GATGGTCTCCTGACCTCCGTTT	130
**Glyceraldehyde 3-phosphate dehydrogenase *(GAPDH)***	CTCTGACTTCAACAGCGACACCC	TCCTCTTGTGCTCTTGCTGGGGC	199

Supplementary Table 1. Sequence of the qPCR primers used in this study. BP: base pairs

NTERT-OKF6 (>2fold): 92 Proteins			
Protein	Log_2 fold change	Protein	Log_2 fold change
**KRT7**	-7.88697	**KRT16**	1.89580
**TUBGCP3**	-5.14004	**KRT6B**	1.90172
**ALDH3A1**	-4.72369	**HOOK1**	1.91723
**HIGD2A**	-4.57925	**DSG3**	1.93726
**TGM2**	-4.10728	**CSTA**	1.98320
**CHD8**	-4.06272	**VSNL1**	2.06534
**ABI2**	-3.93499	**TFPI2**	2.19941
**ACAA2**	-3.90295	**DSC2**	2.25012
**IFIH1**	-3.88169	**PRMT3**	2.25523
**SYNPO**	-3.79531	**DEGS1**	2.26218
**CRIP2**	-3.71403	**LYPD3**	2.36809
**KRT19**	-3.64902	**SERPINB2**	2.38662
**SCPEP1**	-3.60900	**ASMTL**	2.41977
**SNCG**	-3.46860	**KRT24**	2.44271
**NNMT**	-3.44793	**DSC3**	2.44751
**ERCC6L**	-3.35669	**TFAP2A**	2.49504
**SDCBP2**	-3.13375	**ANKRD13A**	2.52221
**GLUL**	-3.10382	**KIF13A**	2.53449
**IKBIP**	-3.03723	**PLA2G4A**	2.59702
**XDH**	-3.02992	**BCAT1**	2.65475
**PACSIN3**	-2.89989	**EPPK1**	2.83843
**TCIRG1**	-2.83860	**MGMT**	2.86854
**TSC22D1**	-2.82578	**PRXL2A**	2.93042
**BST2**	-2.82041	**CLIC3**	2.98070
**EFEMP1**	-2.81649	**SPRR1A**	3.10688
**RACGAP1**	-2.78019	**TOP2A**	3.11843
**CTSL**	-2.77956	**MAPK13**	3.22242
**TNC**	-2.63100	**PLCD1**	3.28008
**CDC42EP4**	-2.60036	**NDUFV3**	3.30896
**GSTM1**	-2.59672	**CRABP2**	3.31282
**HCLS1**	-2.56463	**POF1B**	3.37148
**RAD50**	-2.53215	**THBD**	3.55118
**IFITM3**	-2.52856	**GLTP**	3.56682
**HLA-C**	-2.49365	**SBSN**	3.65983
**SLC16A3**	-2.48634	**CYBRD1**	3.66300
**KIF2A**	-2.39657	**LGALS7**	3.69548
**MCAM**	-2.38714	**FLRT3**	3.81578
**GBP1**	-2.22621	**KRT4**	3.91641
**RIPOR1**	-2.20872	**IL18**	3.96070
**PRODH**	-2.08056	**IVL**	4.03843
**TNFRSF10B**	-1.90141	**LGALSL**	4.15027
**MGAT1**	-1.82522	**CES2**	4.23586
**FN1**	-1.62474	**DSG1**	4.24148
		**ANXA8L1**	4.56315
		**SULT2B1**	4.66493
		**SPRR2B**	4.71912
		**KRTDAP**	4.79373
		**A2ML1**	4.99856
		**MTAP**	5.49187

Reactome pathways	#	fold enrichment	raw P value	Proteins
Formation of the cornified envelope	14	24.03	2.77E-15	DSG3, SPRR2B, DSC3, KRT4, KRT19, IVL, KRT6B, KRT16, CSTA, DSC2, DSG1, KRT7, KRT24, SPRR1A
Keratinization	14	14.29	2.14E-12	DSG3, SPRR2B, DSC3, KRT4, KRT19, IVL, KRT6B, KRT16, CSTA, DSC2, DSG1, KRT7, KRT24, SPRR1A
Developmental Biology	16	3.36	2.11E-05	DSG3, SPRR2B, DSC3, KRT4, KRT19, IVL, KRT6B, KRT16, FLRT3, CSTA, DSC2, DSG1, KRT7, MAPK13, KRT24, SPRR1A

Supplementary Table 2. List of all significantly differentially expressed proteins and their log_2 fold change are reported in the proteomic analysis N/TERT1 vs. OKF6/TERT2.

Clone 15-OKF6 (>2fold): 16 Proteins			
**Protein**	**Log_2 fold change**	**Protein**	**Log_2 fold change**
**IRF6**	-4.57725	**FN1**	0.80091
**PTCD3**	-4.12567	**RIDA**	2.82597
**YEATS2**	-4.05369	**COA7**	3.19941
**RBP1**	-3.77503	**MRPS11**	3.80938
**EHBP1L1**	-3.33396	**TOP2A**	3.97821
**MAP10**	-3.11722	**SIPA1L1**	4.08387
**LGALS7**	-3.05917		
**MAP3K3**	-3.04049		
**KRT23**	-2.90119		
**SYNE2**	-2.78544		

Clone 26-OKF6 (>2fold): 30 Proteins			
**Protein**	**Log_2 fold change**	**Protein**	**Log_2 fold change**
**LGALS7**	-6.165050	**FN1**	0.86196
**TPD52**	-4.712097	**MX2**	2.46612
**IRF6**	-4.394848	**HLA-B**	2.47480
**TPM3**	-4.220051	**SAMHD1**	2.51515
**PYGM**	-3.898468	**MINPP1**	2.54703
**ARAP1**	-3.704416	**ANKRD13A**	2.55059
**ERCC6L**	-3.294552	**BST2**	2.69052
**GEMIN4**	-3.230895	**NT5E**	2.72282
**ASS1**	-3.198145	**RIDA**	2.74668
**INTS2**	-3.043492	**ABAT**	2.90908
**MAP3K3**	-2.977794	**COA7**	3.46315
**SLC25A10**	-1.975633	**OAS1**	3.61069
**KRT13**	-1.728635	**MRPS11**	3.65071
		**MX1**	3.79552
		**IFIT1**	4.25527
		**OAS2**	4.79720
		**KRT3**	4.86407

Reactome pathways	#	fold enrichment	raw P value	Proteins
Interferon alpha/beta signaling	9	95.4	7.25E-16	OAS1, IFIT1, OAS2, IRF6, SAMHD1, MX1, HLA-B, BST2, MX2
Interferon signaling	9	32.61	6.70E-12	OAS1, IFIT1, OAS2, IRF6, SAMHD1, MX1, HLA-B, BST2, MX2
Cytokine Signaling in Immune system	10	8.63	1.09E-07	OAS1, IFIT1, OAS2, IRF6, SAMHD1, MAP3K3, MX1, HLA-B, BST2, MX2
Antiviral mechanism by IFN-stimulated genes	5	44.95	1.09E-07	OAS1, IFIT1, OAS2, MX1, MX2
Interferon gamma signaling	4	31.22	9.19E-06	OAS1, OAS2, IRF6, HLA-B
unclassified	3	0.21	2.02E-05	COA7, ANKRD13A, LGALS7
OAS antiviral response	2	>100	1.04E-04	OAS1, OAS2, IRF6, HLA-B
ISG15 antiviral degranulation	3	30.01	1.52E-04	IFIT1, MX1, MX2

Supplementary Table 3. List of all significantly differentially expressed proteins and their log_2 fold change are reported in the proteomic analysis OKF6/TERT2 vs. clones #15 and #26.

Clone 8-NTERT (>2fold): 69 Proteins			
Protein	Log_2 fold change	Protein	Log_2 fold change
LGALS7	-7.20350	SAMD9	1.75048
S100A9	-7.20046	TGFBI	2.07501
ASS1	-6.49613	B2M	2.17009
CRABP2	-5.61240	STRN4	2.17845
IRF6	-5.45500	SP100	2.20775
PRXL2A	-5.43304	PARP9	2.23000
S100A8	-5.32429	OAS2	2.24215
NAGA	-5.19244	SHFL	2.25079
SPRR2B	-5.16952	NNMT	2.32910
LARS2	-4.60264	TYMP	2.37067
KRTDAP	-4.43477	P3H2	2.37124
SULT2B1	-4.35110	PLCG1	2.41219
DSG1	-4.05767	SIRPA	2.42115
IVL	-3.97754	DYNC1LI1	2.42328
LGALSL	-3.74913	HLA-C	2.46036
KRT4	-3.71414	SAMHD1	2.52797
GLTP	-3.63268	ERF	2.57677
SERPINB1	-3.55599	LGALS3BP	2.63867
NT5C	-3.55359	FAM136A	2.76411
GBP6	-3.54427	CPA4	2.87590
SBSN	-3.41310	ISOC2	2.92397
KRT13	-3.23976	PARP14	2.97346
TPD52	-3.19247	POMP	2.97572
TSTD1	-3.12675	BST2	2.98357
TXNL1	-3.01717	HLA-B	3.01237
CLIC3	-2.93995	PIN4	3.03749
HOOK1	-2.85144	MX1	3.11516
ALOX15B	-2.82851	ISG15	3.22845
LDLR	-2.75993	CD82	3.35660
KRT24	-2.73798	IFITM3	3.55231
PLCD1	-2.35364	MX2	3.63423
PCK2	-2.25037	CST3	3.67524
PSPH	-2.14402	IFIH1	4.63579
		IFIT1	4.73719
		HCLS1	4.88137
		POGZ	5.51111

Clone 10-NTERT (>2fold): 80 Proteins			
Protein	Log_2 fold change	Protein	Log_2 fold change
LGALS7	-9.92161	TGFbI	1.59164
S100A9	-7.11567	GLUL	1.74896
SPRR2B	-5.59424	PRKD2	1.96431
S100A8	-5.48779	TUBGCP3	1.98718
IRF6	-5.38279	DYNC1LI1	2.05977
PRXL2A	-5.07028	AQR	2.13884
SULT2B1	-4.98552	PLCG1	2.25777
KRTDAP	-4.57084	IFIT5	2.26604
MSLN	-4.20912	SP100	2.31100
DSG1	-4.15158	B2M	2.35166
RAB38	-4.12052	EXOC2	2.37689
ASS1	-4.00846	OAS2	2.40769
PLCD1	-3.99994	SIRPA	2.46705
IVL	-3.67956	TYMP	2.51555
SBSN	-3.62328	HLA-C	2.53333
GBP6	-3.58750	POLG	2.55452
TPD52	-3.50781	PARP9	2.58850
KRT4	-3.50587	LGALS3BP	2.61934
GLTP	-3.48481	POMP	2.62617
TPBG	-3.11176	PWP2	2.71520
TXNL1	-3.05724	TCEAL4	2.78543
KRT13	-2.98997	ISOC2	2.88933
TGM1	-2.93689	HIGD2A	2.89495
KRT10	-2.86610	FN1	2.90165
RAB25	-2.85530	PIN4	2.90266
LDLR	-2.81039	SAMHD1	3.00510
ALOX15B	-2.77537	ERF	3.04227
ABCC1	-2.69916	UBE2C	3.06634
HOOK1	-2.69231	NNMT	3.10963
MBOAT2	-2.62034	PARP14	3.20170
CLIC3	-2.60277	RAD50	3.24378
A2ML1	-2.31931	HLA-B	3.35241
BCAT1	-2.30366	ISG15	3.38746
SPRR1A	-2.22283	MX1	3.39074
DSC2	-2.13966	NMI	3.43768
ATP6V0A1	-2.12443	IFITM3	3.72944
PSPH	-1.85559	CST3	4.13453
		MX2	4.30594
		LCP1	4.51717
		TSC22D1	4.68141
		HCLS1	5.00505
		POGZ	5.38042
		IFIT1	5.38061

Reactome pathways	#	fold enrichment	raw P value	Proteins
Interferon Signaling	14	18.62	5.60E-14	IFIT1, OAS2, IRF6, PLCG1, SAMHD1, ISG15, HLA-C, GBP6, MX1, SP100, IFITM3, HLA-B, B2M, MX2
Interferon alpha/beta signaling	10	38.91	3.29E-13	IFIT1, OAS2, IRF6, SAMHD1, ISG15, HLA-C, MX1, IFITM3, HLA-B, MX2
Formation of the cornified envelope	9	18.19	2.80E-09	KRT10, SPRR2B, KRT4, KRT13, TGM1, IVL, DSC2, DSG1, SPRR1A
Cytokine Signaling in Immune system	16	5.07	9.22E-08	IFIT1, OAS2, IRF6, PLCG1, SAMHD1, ISG15, LCP1, HLA-C, GBP6, MX1, SP100, IFITM3, HLA-B, FN, B2M, MX2
Interferon gamma signaling	7	20.05	9.49E-08	OAS2, IRF6, HLA-C, GBP6, SP100, HLA-B, B2M
Keratinization	9	10.81	2.01E-07	KRT10, SPRR2B, KRT4, KRT13, TGM1, IVL, DSC2, DSG1, SPRR1A
Antiviral mechanism by IFN-stimulated genes	6	19.8	8.83E-07	IFIT1, OAS2, PLCG1, ISG15, MX1, MX2
Immune System	24	2.9	1.13E-06	IFIT1, OAS2, IRF6, PLCG1, SAMHD1, ISG15, UBE2C, LCP1, HLA-C, GBP6, MX1, CST3, SP100, S100A9, IFITM3, HLA-B, S100A8, DYNC1LI1, DSG1, FN, B2M, MX2, SIRPA, ATP6V0A1
ISG15 antiviral degranulation	5	18.36	1.07E-05	IFIT1, PLCG1, ISG15, MX1, MX2
Neutrophil degranulation	10	5.45	1.64E-05	HLA-C, CST3, S100A9, HLA-B, S100A8, DYNC1LI1, DSG1, B2M, SIRPA, ATP6V0A1
Endosomal/Vacuolar pathway	3	65.17	2.36E-05	HLA-C, HLA-B, B2M
Nicotinamide salvaging	3	41.16	7.85E-05	NNMT, PARP14, PARP9
unclassified	21	0.55	1.04E-04	PRXL2A, ISOC2, IFIT5, KRTDAP, A2ML1, SBSN, POLG, HOOK1, TSC22D1, TCEAL4, LGALS7, PIN4, NMI, POMP, HCLS1, TPBG, CLIC3, POGZ, SULT2B1, HIGD2A, TXNL1
Antigen Presentation: Folding, assembly and peptide loading of class I MHC	3	30.08	1.83E-04	HLA-C, HLA-B, B2M

Supplementary Table 4. List of all significantly differentially expressed proteins and their log_2 fold change are reported in the proteomic analysis OKF6/TERT2 vs. clones #15 and #26.
